# Genome-wide association study identifies multiple susceptibility loci for glioma

**DOI:** 10.1038/ncomms9559

**Published:** 2015-10-01

**Authors:** Ben Kinnersley, Marianne Labussière, Amy Holroyd, Anna-Luisa Di Stefano, Peter Broderick, Jayaram Vijayakrishnan, Karima Mokhtari, Jean-Yves Delattre, Konstantinos Gousias, Johannes Schramm, Minouk J. Schoemaker, Sarah J. Fleming, Stefan Herms, Stefanie Heilmann, Stefan Schreiber, Heinz-Erich Wichmann, Markus M. Nöthen, Anthony Swerdlow, Mark Lathrop, Matthias Simon, Melissa Bondy, Marc Sanson, Richard S. Houlston

**Affiliations:** 1 Division of Genetics and Epidemiology, The Institute of Cancer Research, London SM2 5NG, UK; 2 Sorbonne Universités UPMC Univ Paris 06, INSERM CNRS, U1127, UMR 7225, ICM, F-75013 Paris, France; 3 Onconeurotek, F-75013 Paris, France; 4 AP-HP, GH Pitié-Salpêtrière, Service de Neurologie 2, F-75013 Paris, France; 5 AP-HP, GH Pitié-Salpêtrière, Laboratoire de neuropathologie R Escourolle, F-75013 Paris, France; 6 Department of Neurosurgery, University of Bonn Medical Center, Sigmund-Freud-Straße 25, 53105 Bonn, Germany; 7 Centre for Epidemiology and Biostatistics, Faculty of Medicine and Health, University of Leeds, Leeds LS2 9JT, UK; 8 Department of Biomedicine, Institute of Human Genetics, University of Bonn, 53127 Bonn, Germany; 9 Division of Medical Genetics, Department of Biomedicine, University of Basel, 4056 Basel, Switzerland; 10 1st Medical Department, University Clinic Schleswig-Holstein, Campus Kiel, House 6, Arnold-Heller-Straße 3, Kiel 24105, Germany; 11 Institute of Clinical Molecular Biology, Christian-Albrechts-University Kiel, Arnold-Heller-Straße 3, Kiel 24105, Germany; 12 Institute of Epidemiology I, Helmholtz Zentrum München, German Research Center for Environmental Health, Ingolstädter Landstraße 1, 85764 Neuherberg, Germany; 13 Institute of Medical Informatics, Biometry and Epidemiology, Chair of Epidemiology, Ludwig-Maximilians-Universität, 81377 Munich, Germany; 14 Division of Breast Cancer Research, The Institute of Cancer Research, Sutton, Surrey SM2 5NG, UK; 15 Foundation Jean Dausset-CEPH, 27 Rue Juliette Dodu, 75010 Paris, France; 16 Génome Québec, Department of Human Genetics, McGill University, Montreal, Quebec, Canada H3A 0G1; 17 Division of Hematology-Oncology, Department of Pediatrics, Dan L. Duncan Cancer Center, Baylor College of Medicine, Houston, Texas 77030, USA

## Abstract

Previous genome-wide association studies (GWASs) have shown that common genetic variation contributes to the heritable risk of glioma. To identify new glioma susceptibility loci, we conducted a meta-analysis of four GWAS (totalling 4,147 cases and 7,435 controls), with imputation using 1000 Genomes and UK10K Project data as reference. After genotyping an additional 1,490 cases and 1,723 controls we identify new risk loci for glioblastoma (GBM) at 12q23.33 (rs3851634, near *POLR3B*, *P*=3.02 × 10^−9^) and non-GBM at 10q25.2 (rs11196067, near *VTI1A*, *P*=4.32 × 10^−8^), 11q23.2 (rs648044, near *ZBTB16*, *P*=6.26 × 10^−11^), 12q21.2 (rs12230172, *P*=7.53 × 10^−11^) and 15q24.2 (rs1801591, near *ETFA*, *P*=5.71 × 10^−9^). Our findings provide further insights into the genetic basis of the different glioma subtypes.

## Abstract (web summary)

Using meta-analysis of previous genome-wide association studies against the latest reference variant databases, this work identifies five new risk loci for glioblastoma and non-glioblastoma type of glioma.

## Graphical abstract

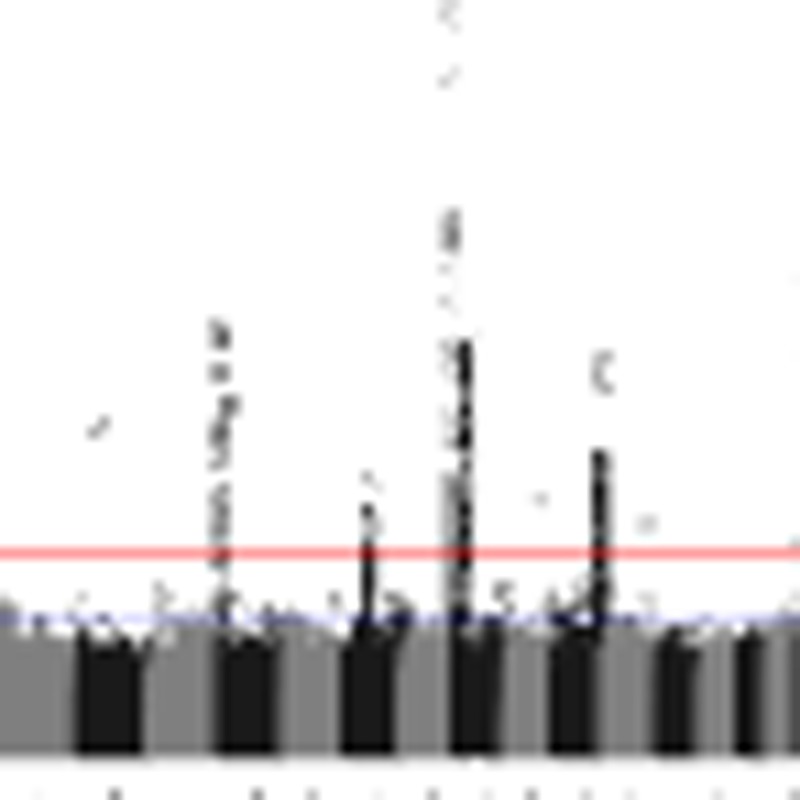

Gliomas account for ∼40% of all primary brain tumours and cause around 13,000 deaths in the United States of America each year^1^. Gliomas are heterogeneous and different tumour subtypes, defined in part by malignancy grade (for example, pilocytic astrocytoma World Health Organization (WHO) grade I, diffuse ‘low-grade' glioma WHO grade II, anaplastic glioma WHO grade III and glioblastoma (GBM) WHO grade IV) can be distinguished^2^. Gliomas are typically associated with a poor prognosis irrespective of clinical care, with the most common type, GBM, having a median overall survival of only 10–15 months^1^.

While the glioma subtypes have distinct molecular profiles resulting from different aetiological pathways^3^, no environmental exposures have, however, consistently been linked to risk except for ionizing radiation, which only accounts for a very small number of cases^1^. Direct evidence for inherited predisposition to glioma is provided by a number of rare inherited cancer syndromes, such as Turcot's and Li–Fraumeni syndromes, and neurofibromatosis^4^. Even collectively, these diseases however account for little of the twofold increased risk of glioma seen in first-degree relatives of glioma patients^5^. Support for polygenic susceptibility to glioma has come from genome-wide association studies (GWASs) that have identified single-nucleotide polymorphisms (SNPs) at eight loci influencing glioma risk—3q26.2 (near *TERC*), 5p15.33 (near *TERT*), 7p11.2 (near *EGFR*), 8q24.21 (near *CCDC26*), 9p21.3 (near *CDKN2A/CDKN2B*), 11q23.3 (near *PHLDB1*), 17p13.1 (*TP53*) and 20q13.33 (near *RTEL1*) (refs 6, 7, 8, 9, 10). Perhaps not surprisingly there is variability in genetic effects on glioma by histology with subtype-specific associations at 5p15.33, 20q13.33 and 7p11.2 for GBM and at 11q23.3 and 8q24 for non-GBM glioma^6^,^7^.

Recovery of untyped genotypes via imputation has enabled fine mapping and refinement of association signals, for example, in identification of rs55705857 as the basis of the 8q24 association signal in glioma^11^. Recently, the use of the 1000 Genomes Project and the UK10K projects as a combined reference panel has been shown to improve accuracy compared with using the 1000 Genomes Project data alone, allowing imputation of alleles with frequencies ∼0.5% to be viable^12^.

Here we report a meta-analysis of four GWASs totalling 4,147 cases and 7,435 controls to identify new glioma susceptibility loci, after imputation using the 1000 Genomes and the UK10K Project data as reference. After genotyping an additional series of 1,490 cases and 1,723 controls we identified new risk loci for GBM at 12q23.33 and non-GBM at 10q25.2, 11q23.2, 12q21.2 and 15q24.2. Our findings provide further insights into the genetic basis of the different glioma subtypes.

## Results

### Association analysis

To identify additional glioma susceptibility loci we conducted a pooled meta-analysis of four GWASs in populations of European ancestry, the UK-GWAS, the French-GWAS, the German-GWAS and the US-GWAS, that were genotyped using either Illumina HumanHap 317, 317+240S, 370Duo, 550, 610 or 1M arrays (Supplementary Table 1). After filtering, the studies provided genotypes on 4,147 cases and 7,435 controls of European ancestry (Supplementary Table 1, Supplementary Fig. 1). Consistent with our previous analysis^6^, quantile–quantile (Q–Q) plots for the German and the US series showed some evidence of inflation (inflation factor based on the 90% least-significant SNPs, *λ*_90_=1.15 and 1.11, respectively), however after correcting for population substructure using principal-component analyses as implemented in Eigenstrat^13^, *λ*_90_ for all four studies was ≤1.05 (combined *λ*_90_=1.05, Supplementary Fig. 2). To achieve consistent and dense genome-wide coverage, we imputed unobserved genotypes at >10 million SNPs using a combined reference panel comprising 1,092 individuals from the 1000 Genomes Project and 3,781 individuals from the UK10K project. Q–Q plots for all SNPs (minor allele frequency (MAF) >0.5%) post-imputation did not show evidence of substantive over-dispersion introduced by imputation after Eigenstrat adjustment (combined *λ*_90_=1.07, *λ*_90_ for individual studies=1.04–1.06; Supplementary Fig. 2).

**Figure 1:**
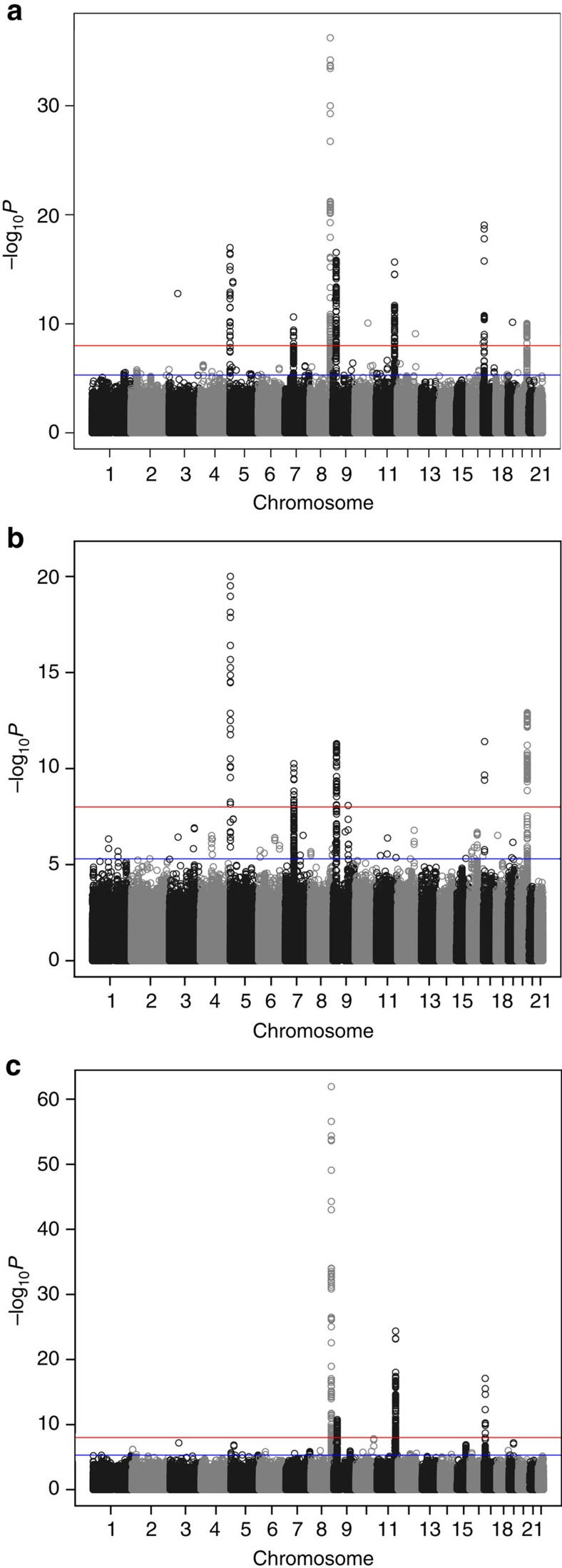
Genome-wide meta-analysis *P* values (–log_10_*P*, *y* axis) plotted against their chromosomal positions (*x* axis). (**a**) All glioma, (**b**) GBM (**c**) non-GBM. The red and blue horizontal lines represent significance thresholds of *P*=5.0 × 10^−8^ and *P*=5.0 × 10^−6^, respectively.

Pooling data from each GWAS into a joint discovery data set, we derived joint odds ratios (ORs) and 95% confidence intervals (CIs) under a fixed-effects model for each SNP with MAF >0.005 and associated per allele Eigenstrat-corrected *P* values. Overall and histology-specific ORs were derived for all glioma, GBM and non-GBM. In the pooled data set, associations at the established risk loci for glioma at 5p15.33, 7p11.2, 8q24.21, 9p21.3, 11q23.3, 17p13.1 and 20q13.33 showed a consistent direction of effect with previously reported studies (*P*<5.0 × 10^−8^, Fig. 1 and Supplementary Table 2). In contrast we found no significant support for the association between rs1920116 near *TERC* (3q26.2) and risk of high-grade glioma recently reported by Walsh *et al*.^10^ (combined *P* value for GBM=0.179; Supplementary Table 2 and Supplementary Fig. 3). While the UK-GWAS and the study of Walsh *et al*. share use of UK 1958 Birth Cohort controls, the other three GWAS we analysed are fully independent.

After filtering at *P*<5.0 × 10^−6^ in either all glioma, GBM or non-GBM, we selected 14 SNPs for follow-up, mapping to distinct loci not previously associated with glioma risk (Fig. 1 and Supplementary Table 2). rs141035288, rs117527984, rs138170678 were not taken forward as there was poor concordance between imputed and sequenced genotypes (Supplementary Table 3), and rs145034266 could not be genotyped as it mapped within a highly repetitive region.

The 10 remaining SNPs underwent replication genotyping in an additional set of 1,490 glioma cases and 1,723 controls (UK replication series, Supplementary Table 4). Meta-analysis was then conducted across discovery and replication stages, with genotype data available on 5,637 cases and 9,158 controls. In the combined analysis five SNPs showed an association with tumour risk, which was genome-wide significant (Table 1)—rs3851634 (12q23.3, *P*_GBM_=3.02 × 10^−9^), rs11196067 (10q25.2, *P*_Non-GBM_=4.32 × 10^−8^), rs648044 (11q23.2, *P*_Non-GBM_=6.26 × 10^−11^), rs12230172 (12q21.2, *P*_Non-GBM_=7.53 × 10^−11^) and rs1801591 (15q24.2, *P*_Non-GBM_=5.71 × 10^−9^). We tested for secondary signals at each locus by adjusting for the sentinel SNP in each region, but found no evidence for independent associations (Supplementary Fig. 4).

**Figure 2:**
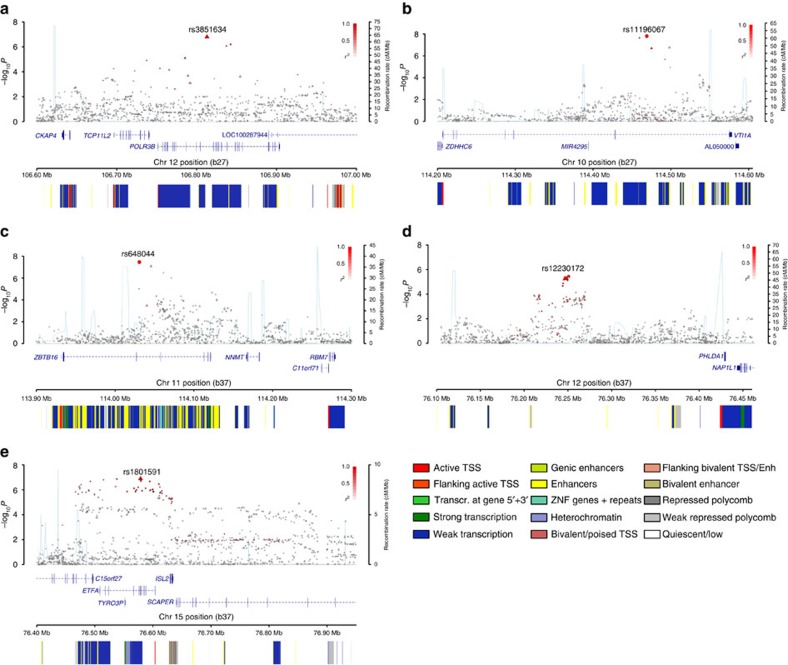
Regional plots of discovery-phase association results, recombination rates and chromatin state segmentation tracks for five glioma-risk loci. Results for: (**a**) 12q23.33, rs3851634 (GBM); (**b**) 10q25.2, rs11196067 (non-GBM); (**c**) 11q23.2, rs648044 (non-GBM); (**d**) 12q21.2 rs12230172 (non-GBM); and (**e**) 15q24.2, rs1801591 (non-GBM). Plots show discovery association results of both genotyped (triangles) and imputed (circles) SNPs in the GWAS samples and recombination rates. The −log_10_ *P* values (*y* axes) of the SNPs are shown according to their chromosomal positions (*x* axes). The lead SNP in each combined analysis is shown as a large circle or triangle (if imputed or directly genotyped, respectively) and is labelled by its rsID. The colour intensity of each symbol reflects the extent of LD with the top genotyped SNP, white (*r*^2^=0) to dark red (*r*^2^=1.0). Genetic recombination rates, estimated using HapMap samples from Utah residents of western and northern European ancestry (CEU), are shown with a light blue line. Physical positions are based on NCBI build 37 of the human genome. Also shown are the relative positions of University of Carolina, Santa Cruz (UCSC) genes and transcripts mapping to the region of association. Genes have been redrawn to show their relative positions; therefore, maps are not to physical scale. Below each plot is a diagram of the exons and introns of the genes of interest, the associated SNPs and the chromatin state segmentation track (ChromHMM) for H1 neural progenitor cells derived from the epigenome roadmap project, as per legend. TSS, transcriptional start sites.

The association signal at 12q23.3 defined by rs3851634 was specific for GBM. The rs3851634 maps to intron 12 of the gene encoding polymerase III, RNA, subunit b (*POLR3B*; Fig. 2a) within a ∼350-kb block of linkage disequilibrium (LD) at 12q23.3, which also contains the genes *CKAP4* and *TCP1L2*. The other four SNP associations defined by rs11196067, rs648044, rs12230172 and rs1801591 were specific to non-GBM glioma. rs11196067 (10q25.2) is located in intron 7 of *VTI1A* (vesicle transport through interaction with t-SNAREs 1A, Fig. 2b). Similarly rs648044 (11q23.2) is also intronic mapping within ZBTB16 (zinc finger and BTB domain-containing protein 16, alias PLZF; Fig. 2c). The rs12230172 (12q21.2) maps within the lincRNA *RP11-114H23.1* and is centromeric to the gene encoding *PHLDA1* (centromeric pleckstrin homology-like domain, family a, MEMBER 1, Fig. 2d). rs1801591 (15q24.2) is responsible for the p.Thr171Ile substitution in *ETFA* (electron transfer flavoprotein, alpha polypeptide gene, which resides within a 500-kb region of LD to which *ISL2*, *TYRO3P* and *SCAPPER* genes also map Fig. 2e).

### Relationship between the new glioma SNPs and tumour profile

To investigate the impact of the new risk SNPs on glioma subtype we examined rs11196067, rs648044, rs12230172, rs1801591 and rs3851634 genotypes in the French case series for which comprehensive histology and molecular phenotyping had been performed (Supplementary Data 1). The GBM SNP rs3851634 was associated with 10q-deleted glioma (*P*=0.016). In the case of non-GBM SNPs rs11196067 showed the strongest association with grade II glioma (*P*=3.2 × 10^−5^) and *TP53* non-mutated glioma (*P*=5.82 × 10^−5^); rs648044 with grade II oligodendroglioma (*P*=0.026) and 10q non-deleted glioma (*P*=0.006); rs1801591 with grade II astrocytoma (*P*=0.001) and *IDH1/IDH2* mutated glioma (*P*=0.005) and rs12230172 with grade II oligodendroglioma (*P*=0.009), *IDH1/IDH2* mutated (*P*=0.009) and 10q non-deleted glioma (*P*=0.003).

### Functional annotation of risk variants

For each of the sentinel risk SNPs at the five risk loci (as well as correlated variants, *r*^2^>0.8) we examined published data^14^,^15^ and made use of the online resources HaploReg v3, RegulomeDB and SeattleSeq for evidence of functionality and regulatory motifs at genomic regions (Supplementary Table 5). rs1801591, which is responsible for the *ETFA* p.Thr171Ile substitution, resides within a highly conserved region of the genome (genomic evolutionary rate profiling (GERP)=5.65) and the amino-acid change is predicted to be damaging (PolyPhen=1). Although rs648044 exhibits low evolutionary conservation (GERP=−9.32) it maps within a strong DNase hypersensitivity site and predicted enhancer/super-enhancer element for multiple tissues including the brain. The region surrounding rs648044 is also predicted to interact with the *ZBTB16* promoter, which combined with alteration of a Pax-5 motif is suggestive of direct functional impact. rs12230172 localizes within a moderately conserved region (GERP=3.41) and occupies promoter histone marks in the brain as well as enhancers predicted to associate with transcriptional start sites for *PHLDA1* and *GLIPR1*. rs11196067 in *VTI1A,* while having a low conservation score (GERP=0.719), occupies enhancer histone marks in embryonic stem cells although not in brain cells. Similarly, rs3851634 maps to a moderately conserved region (GERP=2.37) and occupies enhancer histone marks in 18 organs including the brain.

### eQTL analysis of the five new glioma SNPs

To gain further insight into the functional basis of rs11196067, rs648044, rs12230172, rs1801591 and rs3851634 associations we performed an expression quantitative trait loci (eQTL) analysis using RNA-Seq expression data on 389 low-grade gliomas (LGGs) and 138 GBMs from The Cancer Genome Atlas (TCGA), together with lymphoblastoid cell line RNA-Seq data on 363 samples from GEUVADIS^16^. We examined for an association between SNP genotype and expression of genes mapping within 1 Mb of the sentinel SNP (Supplementary Data 2). After adjusting for multiple testing within each region no statistically significant eQTL was seen for rs11196067, rs12230172, rs1801591 or rs3851634. The strongest association between rs648044 genotype and gene expression was with *ZW10* in LGG (*P*=5.7 × 10^−5^), with the risk allele (T) associated with lower expression, remaining significant after adjustment for multiple testing. To explore the possibility that rs648044 is correlated with a SNP exhibiting a stronger association with *ZW10*, we examined associations with *ZW10* expression in LGG tumours in all SNPs in LD (*r*^2^>0.4) with rs648044. All of the proxy SNPs examined were more weakly associated with *ZW10* than rs648044 (Supplementary Table 6). Following on from these analyses we made use of publically available eQTL mRNA expression array data on adipose tissue, lymphoblastoid cell lines and skin from 856 twins (MuTHER^17^) and 5,311 non-transformed peripheral blood samples using the blood eQTL browser^18^. The risk allele (C) of rs3851634 was associated with significantly lower levels of *POLR3B* (*P*=7.49 × 10^−6^) in peripheral blood analysis with a nominally significant association in skin (*P*=0.0052). The risk allele (T) of rs1801591, was associated with significantly lower *ETFA* levels in peripheral blood (*P*=7.90 × 10^−12^); there was a nominally significant association in MuTHER lymphoblastoid cell lines (*P*=0.037).

### Somatic mutation of newly implicated risk genes in glioma

We examined mutation data from TCGA for evidence of recurrent mutation in genes annotated by the new GWAS signals. Collectively *POLR3B*, *ETFA*, *VTI1A*, *ZBTB16* and *PHLDA1* are altered in 8% (22/286) of LGG as compared with 3% (8/273) of GBM (*P*=0.014, Supplementary Table 7) providing support for these genes having a role in glioma tumorigenesis.

### Individual variance in risk associated with glioma SNPs

To explore the relative contributions of previously reported and newly described loci to glioma risk, we applied the method of Pharoah *et al*.^19^ to eight previously reported SNPs as well as the five new risk SNPs (Supplementary Table 8). The variance in risk attributable to all 12 SNPs is 26%, 27% and 43% for all glioma, GBM and non-GBM, respectively.

### Pathway enrichment of glioma GWAS SNPs

To gain further insights into the biological basis of associations we performed a pathway analysis on GWAS associations in all glioma, GBM and non-GBM. Applying a false discovery rate (FDR) threshold of <0.1 revealed enrichment for 14 pathways in all glioma, 8 in GBM and 9 in non-GBM tumours (Supplementary Table 9). Pathways implicated in GBM tumours primarily include DNA repair and Notch-signalling, whereas for non-GBM tumours pathways were primarily associated with cell-cycle progression and energy metabolism (Supplementary Table 9).

## Discussion

To our knowledge we have performed the largest GWAS of glioma to date, identifying five novel glioma susceptibility loci at 12q23.33, 10q25.2, 11q23.2, 12q21.2 and 15q24.2 and taking the total count of risk loci to 12. Through making use of a combined reference panel from the UK10K and the 1000 Genomes Projects we were able to recover genotypes from ∼8 million SNPs for association analysis, a significant increase from using array SNPs alone. In addition, we have provided further evidence that genetic susceptibility to glioma can be subtype specific, emphasising the importance of searching for histology-specific risk variants.

While deciphering the functional impact of these SNP associations on glioma development requires additional analyses, a number of the genes implicated have relevance to the biology of this cancer *a priori*. As well as participating in regulating insulin-stimulated trafficking of secretory vesicles^20^, *VTI1A* plays a key role in neuronal development and in selectively maintaining spontaneous neurotransmitter release^21^. Intriguingly recent GWAS have identified associations between the *VTI1A* SNPs rs7086803 and lung cancer^22^ and between rs12241008 and colorectal cancer^23^; rs7086803 and rs12241008 are not correlated with each other (*r*^2^=0.22, *D′*=0.72) and are also not correlated with rs11196067 (*r*^2^=0.03/0.00 *D′* =1.00/0.22, respectively), suggesting the existence of multiple risk loci within the region with different tumour specificities.

*ZBTB16* is highly expressed in undifferentiated, multipotential progenitor cells and its expression has been shown to influence resistance to retinoid-mediated re-differentiation in *t*(11;17)(q23;21) acute promyelocytic leukaemia^24^. The BTB domain of *ZBTB16* has transcriptional repression activity and interacts with components of the histone deacetylase complex thereby linking the transcription factor with regulation of chromatin conformation^25^. Although rs648044 lies within an enhancer active in brain and is predicted to interact with the *ZBTB16* promoter, providing an attractive functional basis for the 11q23.2 association through differential ZBTB16 expression, we found a strong association between rs648044 and *ZW10* expression in LGG (*P*=5.7 × 10^−5^). Since *ZW10* plays a role in chromosome segregation^26^ it also represents a plausible candidate for the 11q23.2 association.

We also observed a strong association between *ETFA* expression and rs1801591 in peripheral blood (*P*=7.90 × 10^−12^). *ETFA* participates in mitochondrial fatty acid beta oxidation; shuttling electrons between flavoprotein dehydrogenases and the membrane-bound electron transfer flavoprotein ubiquinone oxidoreductase^27^. Mutations of *ETFA* have been reported to be a cause of recessive glutaric acidaemia IIA (refs 28, 29), which features gliosis. While the p.Thr171Ile change is reported to decrease thermal stability of *ETFA*^30^ thereby providing evidence for a direct functional effect the strong eQTL data is consistent with the functional basis for the 15q24.2 association being mediated through differential expression.

RNA polymerase III (*POLR3B*) is involved in the transcription of small noncoding RNAs and short interspersed nuclear elements, as well as all transfer RNAs^31^. Although mutations in *POLR3B* have been shown to cause recessive hypomyelinating leukoencephalopathy^32^ thus far there is no evidence implicating the gene in the development of glioma. Albeit in peripheral blood there was a strong association between *POLR3B* expression and rs3851634 (*P*=7.49 × 10^−6^), providing a possible functional basis of the 12q23.2 association.

At 12q21.2 rs12230172 maps within *RP11-114H23.1*, a lincRNA of currently unknown function. Although only lying adjacent to *PHLDA1*, the known 11q23.3 association maps to the related gene *PHLDB1*, which is also specific to non-GBM tumours^7^. Although a role for *PHLDA1* in glioma has yet to be established downregulation of *PHLDA1* in neuronal cells has been shown to enhance cell death without Fas induction^33^, additionally *PHLDA1* expression may be involved in regulation of anti-apoptotic effects of *IGF1* (ref. 34).

Intriguingly across all of the four GWAS data sets we analysed we did not replicate the association between rs1920116 (near *TERC*) at 3q26.2 and risk of high-grade glioma recently reported by Walsh *et al*.^10^ (*P*=8.3 × 10^−9^, OR=1.30 versus *P*=0.18, OR=1.06 relative to the G-allele in our GBM data set), despite our study having a similar power to demonstrate a relationship (1,783 GBM cases, 7,435 controls in our study as compared with 1,644 cases, 7,736 controls). It is, however noteworthy that the Walsh *et al*. analysed both anaplastic astrocytoma and GBM. While we could not demonstrate a significant association with either subtype we did see an association between rs1920116 and *TP53*-mutated glioma (*P*=0.016, Supplementary Data 1) suggesting that the association might be restricted to a specific molecularly defined subtype of glioma.

Our findings provide further evidence for an inherited genetic susceptibility to glioma. Future investigation of the genes targeted by the risk SNPs we have identified is likely to yield increased insight into the development of this malignancy. We estimate that the risk loci so far identified for glioma account for 27 and 43% of the familial risk of GBM and non-GBM tumours, respectively, of which 0.8% and 7.6% can be explained by the loci newly reported in this study (Supplementary Table 8). Although the power of our study to detect the major common loci (MAF>0.2) conferring risk ≥1.2 was high (∼80%), we had low power to detect alleles with smaller effects and/or MAF<0.1. By implication, variants with such profiles probably represent a much larger class of susceptibility loci for glioma because of the truly small effect sizes or submaximal LD with tagging SNPs. Thus, it is probable that a large number of variants remain to be discovered. In addition, as we have recently shown, stratified analysis of glioma by molecular profile may lead to the discovery of additional subtype-specific risk variants. However, such subtype analyses can increase the statistical burden of adjusting for multiple testing. For example, if applying an additional Bonferroni correction for GBM and non-GBM subtypes, the rs11196067 (*VTI1A*) association at *P*=8.64 × 10^−8^ would not be declared genome-wide significant. An issue in future subtype analyses of glioma will therefore be to have sufficient study power to mitigate type II error given the additional constraints of multiple testing. Further efforts to expand the scale of GWAS meta-analyses through international consortia and increasing the number of SNPs taken forward to large-scale replication will be required to address this challenge.

## Methods

### Ethics

Collection of blood samples and clinico-pathological information from patients and controls was undertaken with informed consent and relevant ethical review board approval in accordance with the tenets of the Declaration of Helsinki. Ethical committee approval for this study was obtained from relevant study centres (UK: South East Multicentre Research Ethics Committee (MREC) and the Scottish Multicentre Research Ethics Committee; France: APHP Ethical Committee-CPP (comité de Protection des Personnes); Germany: Ethics Commission of the Medical Faculty of the University of Bonn; and USA: University of Texas MD Anderson Cancer Institutional Review Board).

### Genome-wide association studies

We used GWAS data previously generated on four non-overlapping case–control series of Northern European ancestry, which have been the subject of previous studies^6^,^7^; summarized in Supplementary Table 1. Briefly, the UK-GWAS was based on 636 cases (401 males; mean age 46 years) ascertained through the INTERPHONE study^35^. Individuals from the 1958 Birth Cohort (*n*=2,930) served as a source of controls. The US-GWAS was based on 1,281 cases (786 males; mean age 47 years) ascertained through the MD Anderson Cancer Center, Texas, between 1990 and 2008. Individuals from the Cancer Genetic Markers of Susceptibility (CGEMS, *n*=2,245) studies served as controls^36^,^37^. The French-GWAS study comprised 1,495 patients with glioma ascertained through the Service de Neurologie Mazarin, Groupe Hospitalier Pitié-Salpêtrière Paris. The controls (*n*=1,213) were ascertained from the SU.VI.MAX (SUpplementation en VItamines et MinerauxAntioXydants) study of 12,735 healthy subjects (women aged 35–60 years; men aged 45–60 years)^38^. The German-GWAS comprised 880 patients who underwent surgery for a glioma at the Department of Neurosurgery, University of Bonn Medical Center, between 1996 and 2008. Control subjects were taken from three population studies: KORA (Co-operative Health Research in the Region of Augsburg; *n*=488) (ref. 39); POPGEN (Population Genetic Cohort; *n*=678) (ref. 40) and from the Heinz Nixdorf Recall study (*n*=380) (ref. 41).

### Replication genotyping

For replication we made use of DNA from 1,490 glioma cases recruited to an ongoing UK study of primary brain tumours (National Brain Tumour Study). Controls were healthy individuals that had been recruited to the National Study of Colorectal Cancer Genetics^42^ and the GEnetic Lung CAncer Predisposition Study^43^. All cases and controls were UK residents and had self-reported European ancestry. Controls reported no personal history of cancer at the time of ascertainment. Genotyping of rs76178334, rs4432939, rs182521816, rs12780046, rs11196067, rs648044, rs12230172, rs3851634, rs1801591 and rs78543262 was performed using competitive allele-specific PCR KASPar chemistry (LGC, Hertfordshire, UK, primer sequences detailed in Supplementary Table 10). Conditions used are available on request. Call rates for SNP genotypes were >95%. To ensure quality of genotyping in all assays, at least two negative controls and 1–10% duplicates (showing a concordance >99%) were genotyped. For SNPs with MAF<5%, at least two known heterozygotes were included per genotyping plate, to aid clustering.

### Statistical and bioinformatic analysis

Data were imputed for all scans for over 10 million SNPs using IMPUTE2 v2.3.0 (ref. 44) software and the 1000 Genomes Project (Phase 1 integrated release 3, March 2012 (ref. 45)) and the UK10K data (ALSPAC, EGAS00001000090/EGAD00001000195, and TwinsUK, EGAS00001000108/EGAD00001000194, studies only) as reference panels (Supplementary Table 1). Genotypes were aligned to the positive strand in both imputation and genotyping. Imputation was conducted separately for each scan in which before imputation each GWAS data set was pruned to a common set of 425,190 SNPs. Poorly imputed SNPs defined by an information score (Is) <0.70 and Hardy–Weinberg equilibrium *P*<1.0 × 10^−5^ were excluded from the analyses. Tests of association between imputed SNPs and glioma was performed under a probabilistic dosage model in SNPTEST v2.5 (ref. 46).

Eigenvectors for the GWAS data sets were inferred using *smartpca* (part of EIGENSOFTv2.4 (refs 13, 47)) using ∼100,00 ld-pruned SNPs. Eigenstrat adjustment was carried out in SNPTEST by including the first 10 eigenvectors as covariates. The adequacy of the case–control matching and possibility of differential genotyping of cases and controls was evaluated using Q–Q plots of test statistics. The inflation factor *λ* was based on the 90% least-significant SNPs as previously advocated^48^. Testing for secondary signals was carried out in SNPTEST, adjusting for the sentinel SNP using the ‘-condition_on' option. Visualization of population ancestry was carried out in *smartpca* by projecting query samples onto eigenvectors inferred from the 1000 Genomes Project populations (Supplementary Fig. 1). Meta-analysis of GWAS data sets under a fixed-effects model was undertaken in META v1.6 (ref. 49) using the inverse-variance approach. Cochran's *Q*-statistic to test for heterogeneity and the *I*^*2*^ statistic to quantify the proportion of the total variation due to heterogeneity were calculated^50^. *P*_het_ values <0.05 are considered characteristic of large heterogeneity^50^. In addition, analyses stratified by glioma tumour histology and molecular characteristics were performed. All statistical *P* values were two sided.

Estimates of individual variance in risk associated with glioma-risk SNPs was carried out using the method described in Pharoah *et al*.^19^ assuming the familial risk of glioma to be 1.77 (ref. 51). Briefly, for a single allele (*i*) of frequency *p*, relative risk *R* and ln risk *r*, the variance (*V*_*i*_) of the risk distribution due to that allele is given by:


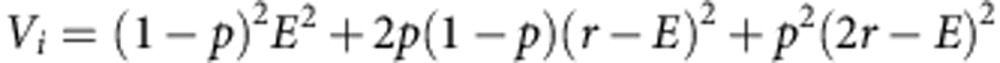


Where *E* is the expected value of *r* given by:


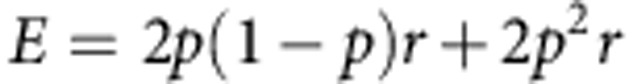


For multiple risk alleles the distribution of risk in the population tends towards the normal with variance:


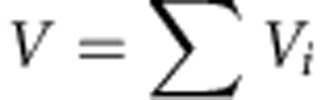


The total genetic variance (*V*) for all susceptibility alleles has been estimated to be √1.77. Thus the fraction of the genetic risk explained by a single allele is given by:


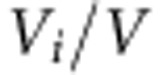


LD metrics were calculated in vcftools v0.1.12b (ref. 52) using UK10K data and plotted using visPIG (ref. 53). LD blocks were defined on the basis of HapMap recombination rate (cM/Mb) as defined using the Oxford recombination hotspots and on the basis of distribution of confidence intervals defined by Gabriel *et al*.^54^

SNPs were annotated for putative functional effect using RegulomeDB^55^, HaploReg v3 (ref. 56) and SeattleSeq Annotation 138 (ref. 57). These servers make use of data from ENCODE^58^, GERP^59^ conservation metrics, combined annotation-dependent depletion (CADD)^60^ scores and PolyPhen 2 (ref. 61) scores. We searched for overlap of associated SNPs with enhancers defined by the FANTOM5 enhancer atlas^15^, annotating by ubiquitous enhancers as well as enhancers specifically expressed in astrocytes, neurons, neuronal stem cells and brain tissue. Similarly, we searched for overlap with ‘super-enhancer' regions as defined by Hnisz *et al*.^14^, restricting analysis to U87 GBM cells, astrocyte cells and brain tissue. We additionally made use of 15-state chromHMM data from H1-derived neuronal progenitor cells available from the Epigenome roadmap project^62^. Mutation data in LGG and GBM tumours from TCGA was assessed using the cBioPortal for cancer genomics^63^.

To search for biological pathways enriched for glioma SNP associations we made use of Improved Gene Set Enrichment Analysis for Genome-wide Association Study (i-GSEA4GWAS v1.1) (ref. 64). SNPs up to 5 kb upstream and downstream of a given gene were mapped to that gene, with the maximum *P* value of all SNPs mapping to a gene used to represent the gene. Gene sets used were: canonical pathways, gene ontology (GO) biological process, GO molecular function, GO cellular component. As recommended we applied an FDR cutoff of <0.10 on all reported gene sets. In the case of multiple identical pathways, that with the lower FDR value is retained.

### Imputation concordance assessment

The fidelity of imputation as assessed by the concordance between imputed and directly genotyped SNPs was examined in 192 cases and 187 controls from the UK-GWAS discovery series (Supplementary Table 3). Targeted sequencing for the SNPs rs141035288, rs117527984, rs76178334, rs4432939, rs182521816, rs138170678, rs145034266, rs12780046, rs11196067, rs648044, rs12230172 and rs78543262 was performed by Sanger on an ABI3700 analyser (Applied Biosystems; Supplementary Table 10, conditions are available on request). For SNPs with MAF <0.05, samples were included to ensure at least 10 predicted heterozygotes were sequenced. Imputed genotypes were considered for concordance assessment if exhibiting probability >0.9.

### Tumour genotyping

Tumour samples were available from a subset of the patients ascertained through the Service de Neurologie Mazarin, Groupe Hospitalier Pitié-Salpêtrière Paris. Tumours were snap frozen in liquid nitrogen and DNA was extracted using the QIAmp DNA minikit, according to the manufacturer's instructions (Qiagen, Venlo, LN, USA). DNA was analysed for large-scale copy number variation by CGH array as previously described^65^,^66^. In the cases not analysed by CGH array, 9p, 10q, 1p and 19q status was assigned using PCR microsatellites, and *EGFR* amplification and *CDKN2A-p16-INK4a* homozygous deletion by quantitative PCR. *IDH1*, *IDH2* and *TERT* promoter mutation status was determined by sequencing as previously described^67^,^68^.

### Expression quantitative trait loci analysis

To examine the relationship between SNP genotype and gene expression, we made use of tumour RNA sequence data and blood Affymetrix 6.0 SNP Array data for 389 low-grade and 138 GBM tumours of European ancestry from TCGA (accession number phs000178.v9.p8), as well as RNA sequence data from lymphoblastoid cells (GEUVADIS project^16^) and genotype data for 363 European individuals from the 1000 Genomes Project^45^. Sequence reads from downloaded FASTQ files were aligned to the human hg19 reference genome and GRCh37 Ensembl transcriptome using TopHat v2.0.7 and Bowtie v2.0.6. Read counts per gene were generated for 62,069 Ensembl genes using featureCounts^69^ as part of the Rsubread Bioconductor package^70^. For TCGA samples, European ancestry was assessed through visualization of clustering with CEU samples after principal components analysis (data not shown). Untyped genotypes were imputed from the Affymetrix 6 array using similar methods to those discussed previously. Genotypes with probability >0.9 were taken forward for eQTL analysis. The association between SNP and gene expression was quantified using the Kruskal–Wallis trend test.

We additionally queried publically available eQTL mRNA expression data using MuTHER, and the Blood eQTL browser. MuTHER contains expression adipose tissue, lymphoblastoid cells and skin expression data from 856 healthy twins^17^. rs500629 was used as a proxy for rs648044 (*r*^2^=0.52, *D′*=0.85). The blood eQTL browser contains expression data from 5,311 non-transformed peripheral blood samples^18^. Putative eQTLs were thresholded at FDR <0.1.

## Additional information

**How to cite this article:** Kinnersley, B. *et al*. Genome-wide association study identifies multiple susceptibility loci for glioma. *Nat. Commun.* 6:8559 doi: 10.1038/ncomms9559 (2015).

## Supplementary Material

Supplementary Information Supplementary Figures 1-4, Supplementary Tables 1-10 and Supplementary References

Supplementary Data 1 Unadjusted association between glioma risk and SNP genotype stratified by tumour histology and molecular features in the French case-control series.

Supplementary Data 2 eQTL analysis of rs11196067, rs648044, rs12230172, rs1801591 and rs3851634.

## Figures and Tables

**Table 1:** Association between SNP and glioma risk in discovery and replication data sets for rs11196067, rs648044, rs12230172, rs3851634 and rs1801591.

	**MAF**			**All glioma**		**GBM**		**Non-GBM**	
**SNP**	**Case**	**Control**	**Study**	***P***	**OR (95% CI)**	***P***	**OR (95% CI)**	***P***	**OR (95% CI)**
rs111696067	0.38	0.41	FRE	5.00 × 10^−5^	0.79 (0.71–0.89)	0.26	0.91 (0.78–1.07)	2.54 × 10^−6^	0.74 (0.66–0.84)
(*VTI1A*)			GER	0.44	0.95 (0.83–1.08)	0.88	1.01 (0.86–1.19)	0.15	0.88 (0.75–1.04)
10q25.2			UK	0.012	0.85 (0.75–0.96)	0.34	0.91 (0.76–1.10)	8.11 × 10^−3^	0.81 (0.69–0.95)
A/**T**			USA	0.016	0.88 (0.80–0.98)	0.097	0.90 (0.79–1.02)	0.033	0.87 (0.76–0.99)
			Replication	0.56	0.97 (0.88–1.07)	0.91	0.99 (0.89–1.11)	0.33	0.93 (0.81–1.07)
			Combined	4.32 × 10^−6^	0.89 (0.85–0.93)	0.11	0.95 (0.89–1.01)	4.32 × 10^−8^	0.84 (0.79–0.89)
rs648044	0.40	0.38	FRE	0.019	1.15 (1.02–1.30)	0.93	0.99 (0.83–1.18)	1.76 × 10^−3^	1.23 (1.08–1.41)
(*ZBTB16*)			GER	0.043	1.16 (1.00–1.34)	0.39	1.08 (0.90–1.30)	0.016	1.25 (1.04–1.50)
11q23.2			UK	0.78	1.02 (0.89–1.16)	0.044	0.82 (0.67–0.99)	0.037	1.20 (1.01–1.42)
C/**T**			USA	0.088	1.10 (0.99–1.23)	0.62	0.97 (0.86–1.09)	1.02 × 10^−3^	1.27 (1.10–1.46)
			Replication	0.97	1.08 (0.97–1.19)	0.59	0.97 (0.86–1.09)	4.16 × 10^−4^	1.29 (1.12–1.48)
			Combined	5.29 × 10^−4^	1.10 (1.04–1.16)	0.32	0.97 (0.90–1.03)	6.26 × 10^−11^	1.25 (1.17–1.34)
rs12230172	0.45	0.46	FRE	0.054	0.90 (0.81–1.00)	0.72	1.03 (0.88–1.20)	4.40 × 10^−3^	0.84 (0.74–0.95)
(intergenic)			GER	0.043	0.88 (0.77–1.00)	0.84	0.98 (0.84–1.16)	2.17 × 10^−3^	0.78 (0.66–0.91)
12q21.2			UK	0.44	0.95 (0.84–1.09)	0.77	0.97 (0.85–1.11)	0.42	0.94 (0.80–1.10)
G/**A**			USA	0.30	0.95 (0.86–1.05)	0.55	1.04 (0.92–1.18)	0.018	0.85 (0.75–0.97)
			Replication	1.84 × 10^−6^	0.79 (0.70–0.86)	7.00 × 10^−3^	0.85 (0.76–0.96)	3.59 × 10^−8^	0.67 (0.58–0.77)
			Combined	1.57 × 10^−6^	0.88 (0.84–0.93)	0.22	0.96 (0.91–1.02)	7.53 × 10^−11^	0.81 (0.76–0.86)
rs3851634	0.27	0.30	FRE	0.053	0.89 (0.79–1.00)	0.020	0.81 (0.69–0.97)	0.25	0.93 (0.81–1.06)
(*POLR3B*)			GER	0.18	0.91 (0.73–1.04)	0.12	0.87 (0.73–1.04)	0.59	0.95 (0.80–1.14)
12q23.3			UK	0.058	0.88 (0.60–0.89)	1.56 × 10^−3^	0.73 (0.60–0.89)	0.92	1.01 (0.85–1.20)
T/**C**			USA	2.84 × 10^−4^	0.81 (0.73–0.91)	7.21 × 10^−4^	0.79 (0.68–0.90)	0.021	0.84 (0.73–0.98)
			Replication	0.022	0.88 (0.79–0.98)	5.00 × 10^−3^	0.83 (0.74–0.95)	0.57	0.96 (0.83–1.11)
			Combined	4.07 × 10^−7^	0.87 (0.82–0.92)	3.02 × 10^−9^	0.81 (0.76–0.87)	0.037	0.93 (0.87–1.00)
rs1801591	0.10	0.09	FRE	6.67 × 10^−3^	1.32 (1.08–1.61)	0.29	1.17 (0.87–1.58)	2.51 × 10^−3^	1.40 (1.13–1.74)
(*ETFA*)			GER	0.037	1.25 (1.01–1.53)	0.17	1.20 (0.93–1.56)	0.052	1.31 (1.00–1.72)
15q24.2			UK	0.44	1.08 (0.88–1.33)	0.93	0.99 (0.73–1.33)	0.23	1.17 (0.90–1.53)
G/**A**			USA	0.016	1.23 (1.04–1.46)	0.97	1.00 (0.80–1.24)	5.13 × 10^−5^	1.56 (1.26–1.94)
			Replication	0.16	1.13 (0.95–1.33)	0.89	1.01 (0.83–1.23)	0.013	1.31 (1.06–1.63)
			Combined	2.75 × 10^−5^	1.20 (1.10–1.30)	0.32	1.06 (0.95–1.18)	5.71 × 10^−9^	1.36 (1.23–1.51)

ORs derived with respect to the minor allele, highlighted in bold. The SNPs rs3851634 and rs1801591 were directly genotyped while rs1196067, rs648044 and rs12230172 were imputed with imputation information scores (Is) of 0.99, 0.87 and 1.00, respectively. Sample sizes in the individual data sets are as follows: FRE (French-GWAS), 1,423 cases and 1,190 controls; GER (German-GWAS), 846 cases and 1,310 controls; UK (UK-GWAS), 631 cases and 2,699 controls; USA (USA-GWAS), 1,247 cases and 2,236 controls; replication, 1,490 cases and 1,723 controls; combined, 5,637 cases and 9,158 controls. MAF, minor allele frequency in discovery series.
