# Supplementary material for: Genome-wide association study identifies multiple susceptibility loci for glioma
Source: Nat Commun. 2015 Oct 1;6:8559. doi: 10.1038/ncomms9559 (PMC4600760; doi:10.1038/ncomms9559)
Supplement: Supplementary Information — Supplementary Figures 1-4, Supplementary Tables 1-10 and Supplementary References [file ncomms9559-s1.pdf]

Supplementary Figures

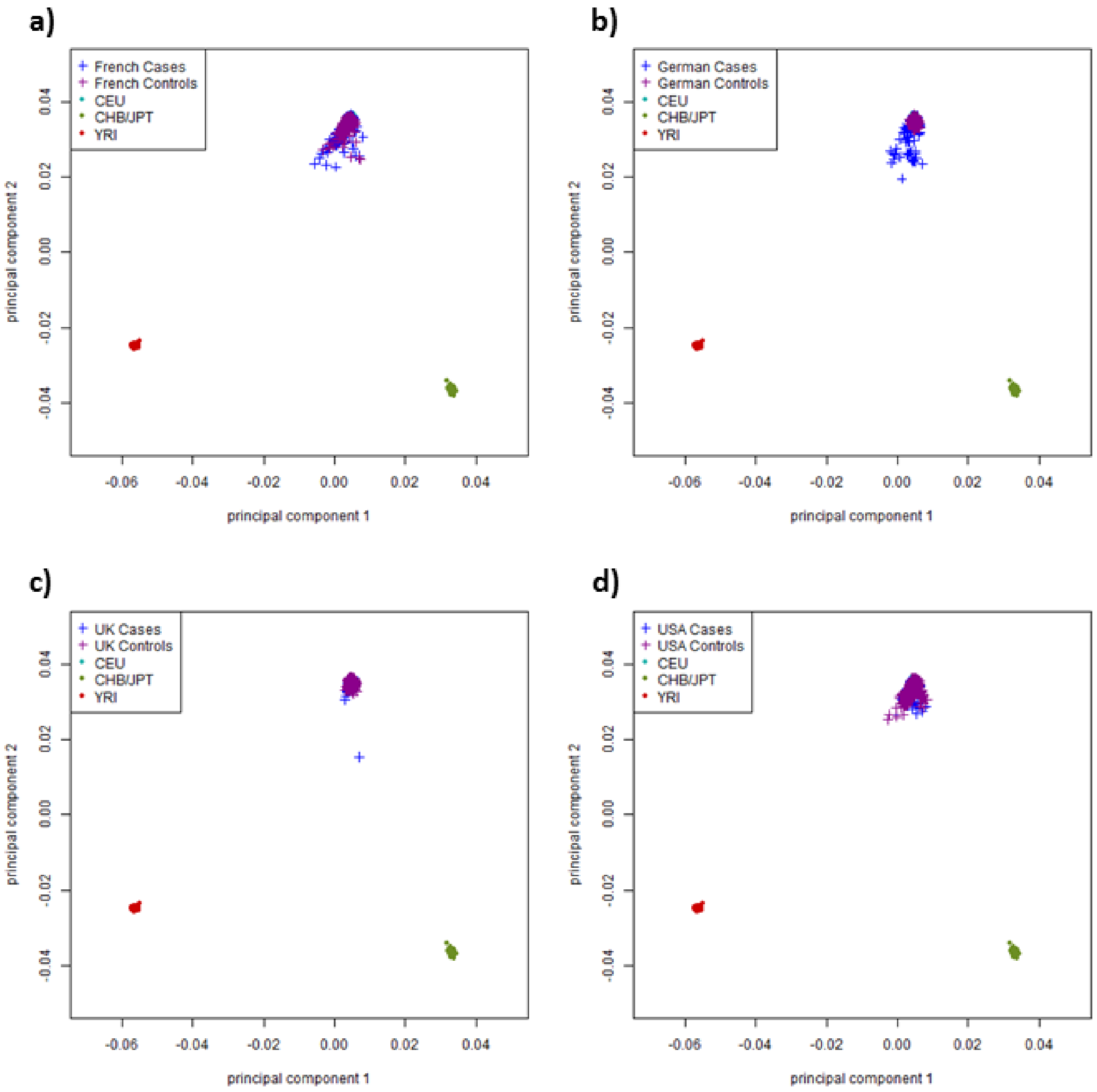

Supplementary Figure 1: PCA plots of HapMap populations with the four GWAS datasets: a) French-GWAS; b) German-GWAS; c) UK-GWAS; d) USA-GWAS. Cases and controls are plotted as purple and blue crosses respectively. Red, green and teal circles respectively represent HapMap YRI, CHB/JPT and CEU populations.

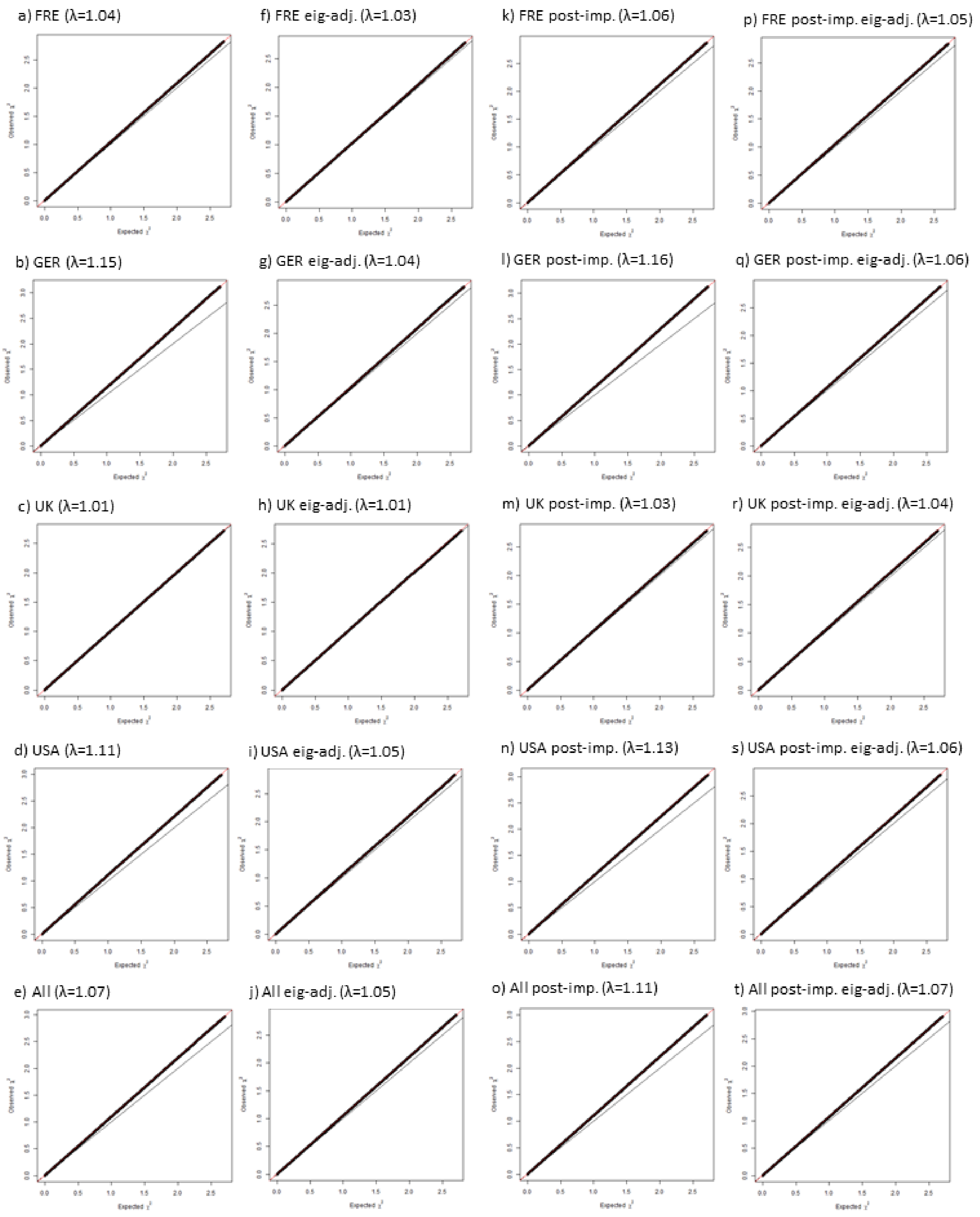

Supplementary Figure 2: Q-Q plots of association statistics for the four Glioma GWAS datasets: (a-e), pre-imputation; (f-j), pre-imputation eigenstrat-adjusted; (k-o), post-imputation; (p-t), post-imputation eigenstrat adjusted. FRE, French-GWAS; GER, German-GWAS; UK, UK-GWAS; USA, USA-GWAS; All, combined meta-analysis.

a) 3q26.2 , rs1920116 (all glioma)

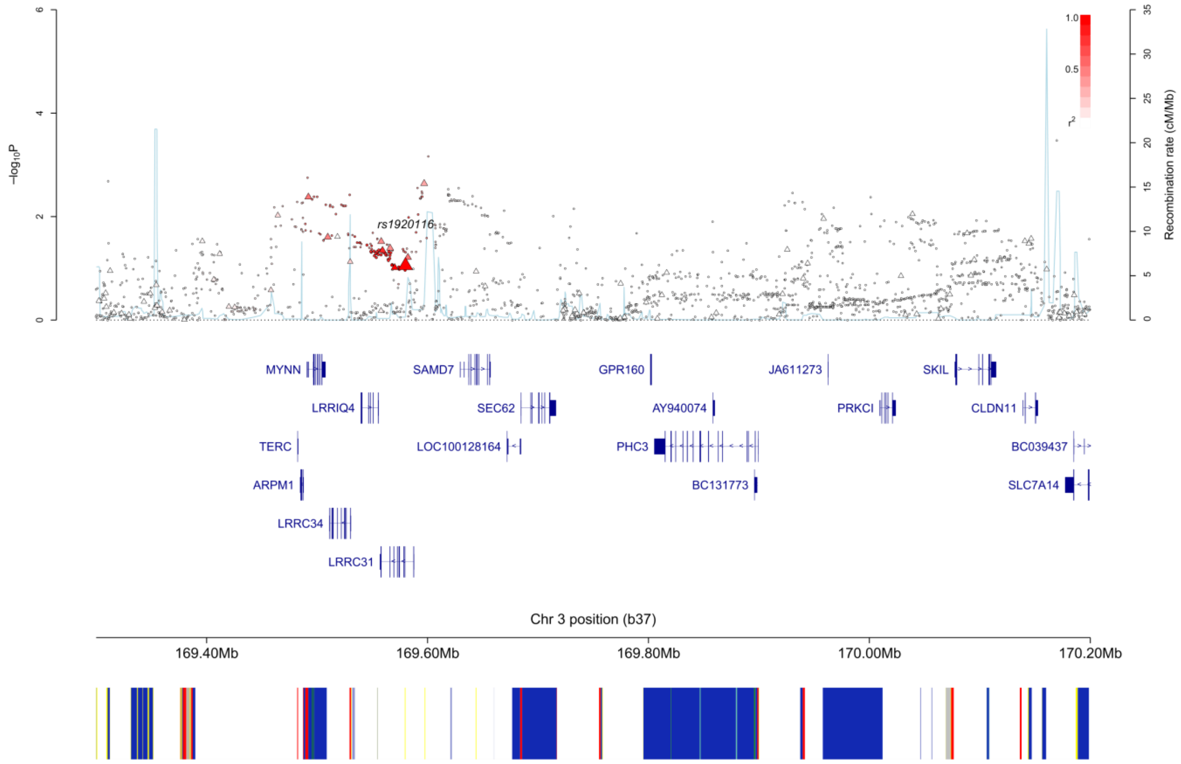

b) 3q26.2 , rs1920116 (GBM)

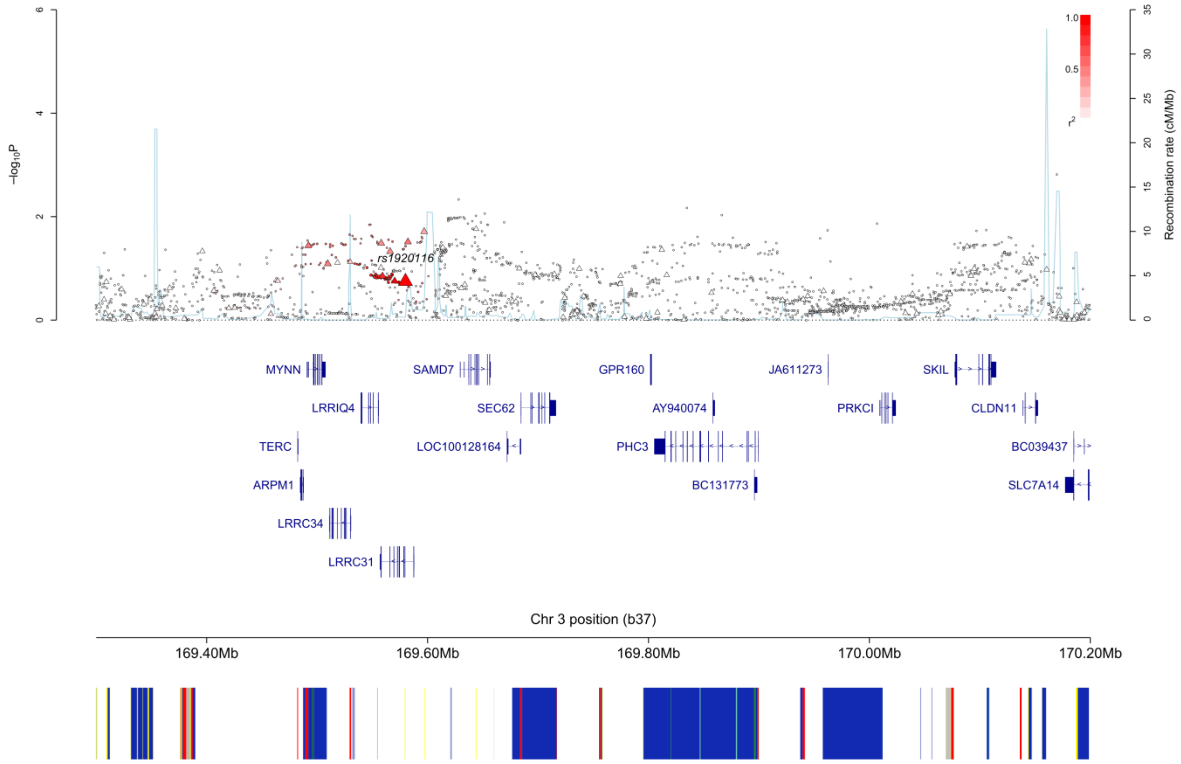

c) 3q26.2 , rs1920116 (non GBM)

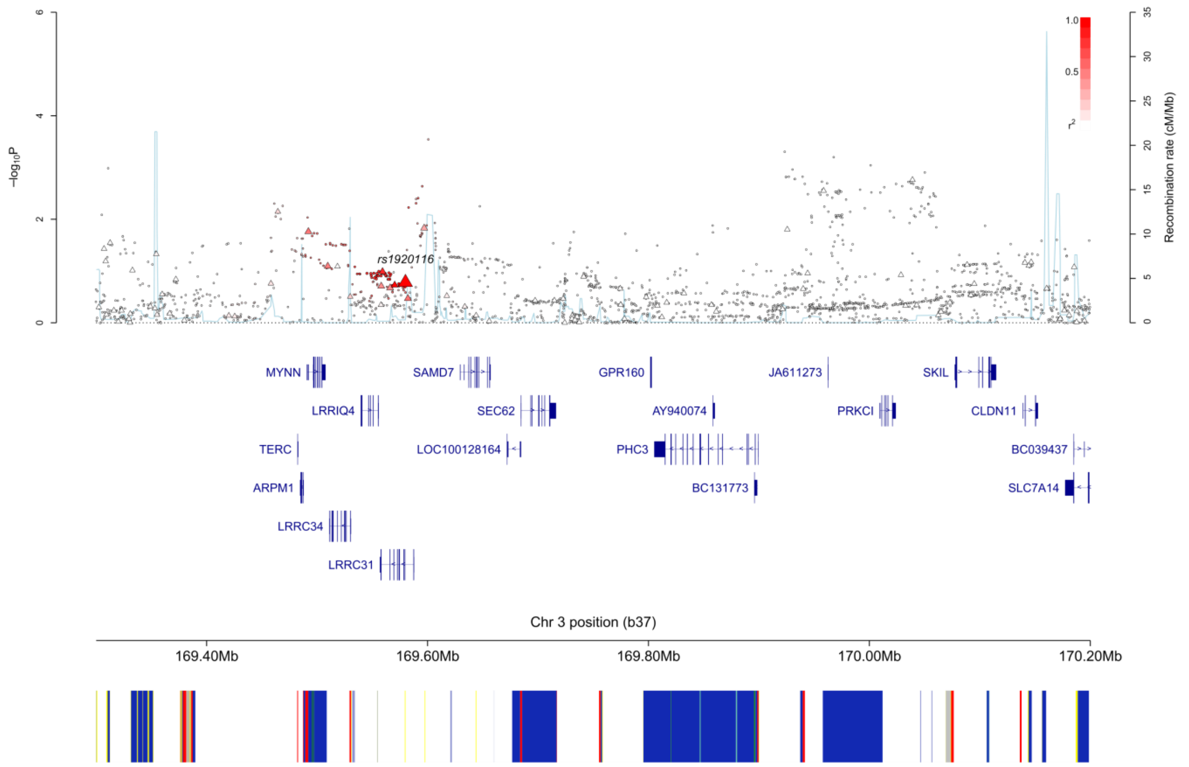

d)

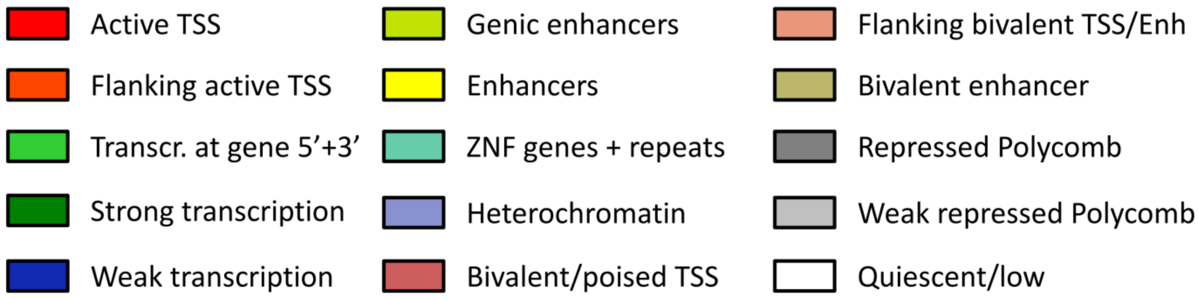

Supplementary Figure 3: Regional plots of association results, recombination rates and chromatin state segmentation tracks for published glioma risk locus at 3q26.2 (rs1920116). Results for: (a) All Glioma; (b) GBM; (c) non GBM). Plots show association results of both genotyped (triangles) and imputed (circles) SNPs in the GWAS samples and recombination rates. $-\log_{10} P$ values (y axes) of the SNPs are shown according to their chromosomal positions (x axes). rs1920116 in each combined analysis is shown as a large circle or triangle (if imputed or directly genotyped respectively) and is labeled by its rsID. The color intensity of each symbol reflects the extent of LD with the top genotyped SNP, white ($r^2 = 0$) through to dark red ($r^2 = 1.0$). Genetic recombination rates, estimated using HapMap samples from Utah residents of western and northern European ancestry (CEU), are shown with a light blue line. Physical positions are based on NCBI build 37 of the human genome. Also shown are the relative positions of genes and transcripts mapping to the region of association. Genes have been redrawn to show their relative positions; therefore, maps are not to physical scale. Below each plot is a diagram of the exons and introns of the genes of interest, the associated SNPs and the chromatin state segmentation track (ChromHMM) for H1 neural progenitor cells derived from the epigenome roadmap project. (d) Legend file depicting chromatin states for chromHMM track.

a) 12q23.33, rs3851634 (GBM)

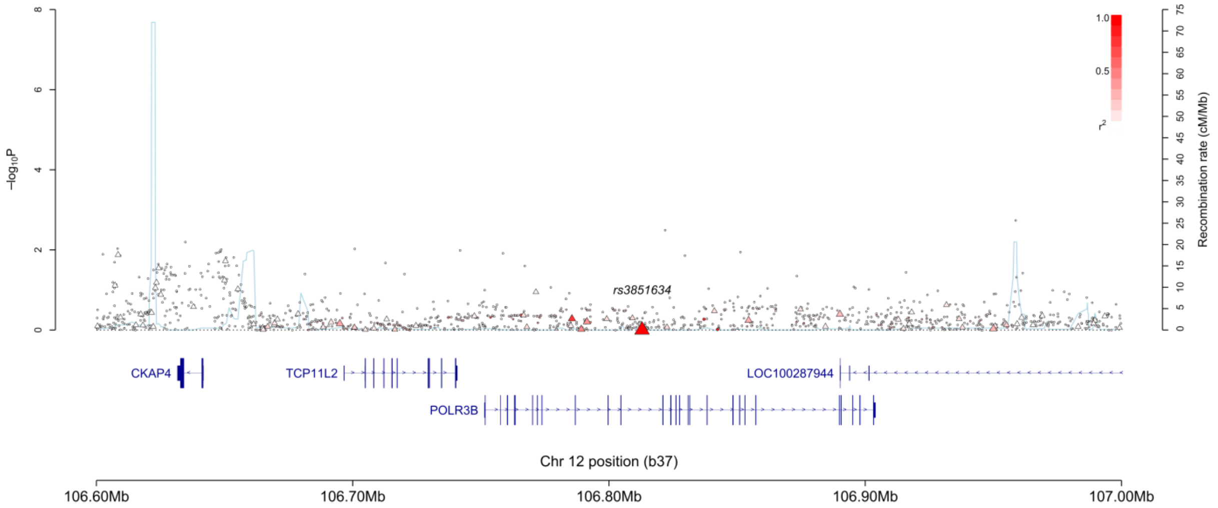

b) 10q25.2, rs11196067 (non GBM)

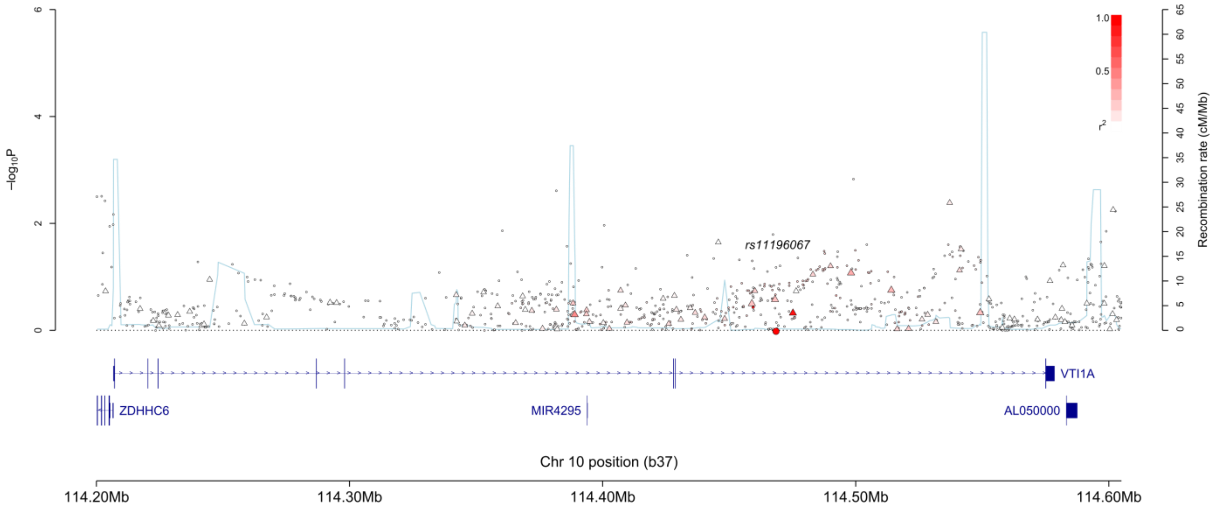

c) 11q23.2, rs648044 (non GBM)

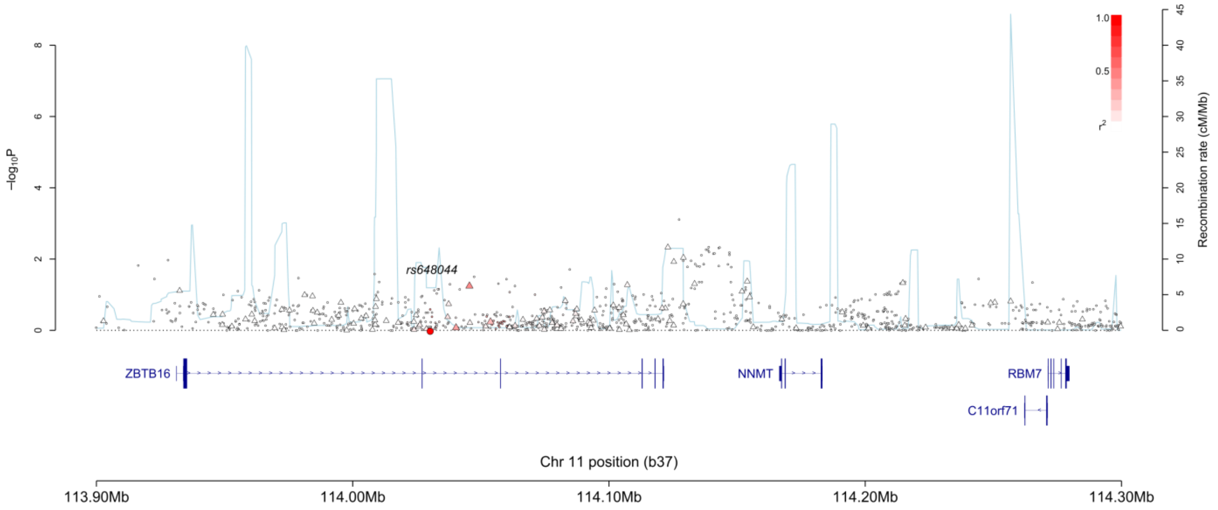

d) 12q21.2 rs12230172 (non GBM)

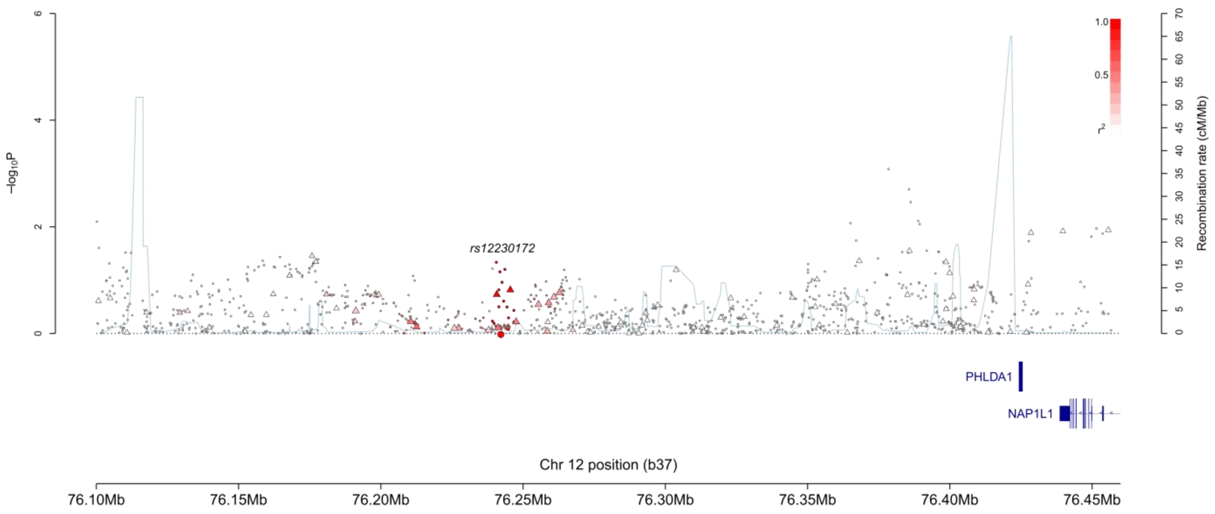

e) 15q24.2, rs1801591 (non GBM)

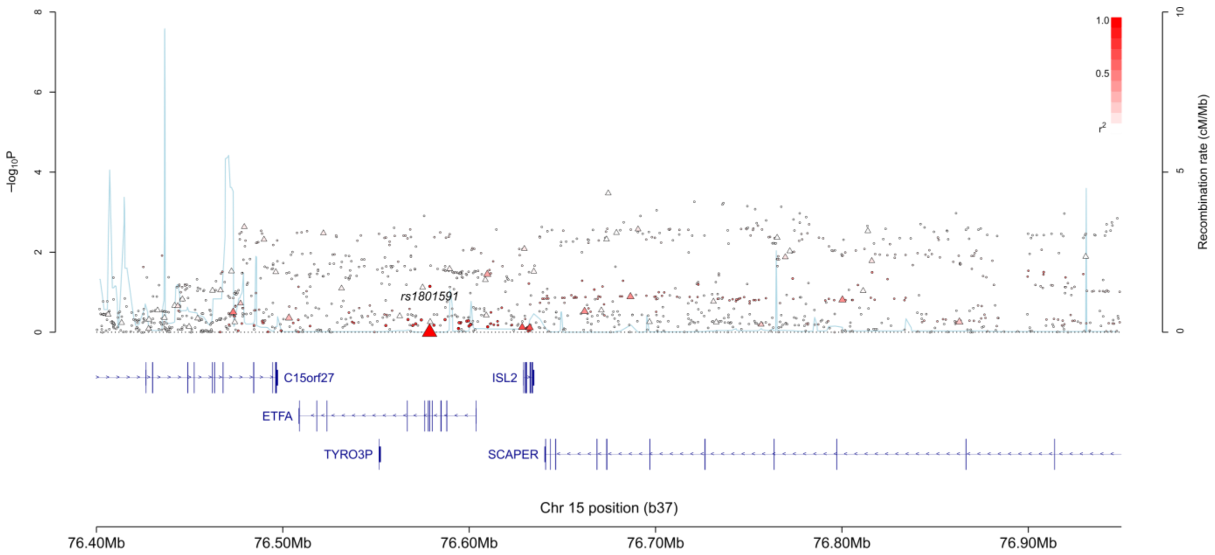

Supplementary Figure 4: Regional plots of discovery-phase association results, recombination rates and chromatin state segmentation tracks for five glioma risk loci after adjusting for the lead SNP at each locus. Results for: (a) 12q23.33, rs3851634 (GBM); (b) 10q25.2, rs11196067 (non GBM); (c) 11q23.2, rs648044 (non GBM): (d) 12q21.2 rs12230172 (non GBM) and (e) 15q24.2, rs1801591 (non GBM). Plots show association results of both genotyped (triangles) and imputed (circles) SNPs in the GWAS samples and recombination rates. $-\log_{10}$ *P* values (*y* axes) of the SNPs are shown according to their chromosomal positions (*x* axes). The lead SNP being adjusted is shown as a large circle or triangle (if imputed or directly genotyped) and is labeled by its rsID. The color intensity of each symbol reflects the extent of LD with the top genotyped SNP, white (r^2 = 0) through to dark red (r^2 = 1.0). Genetic recombination rates, estimated using HapMap samples from Utah residents of western and northern European ancestry (CEU), are shown with a light blue line. Physical positions are based on NCBI build 37 of the human genome. Also shown are the relative positions of UCSC genes and transcripts mapping to the region of association. Genes have been redrawn to show their relative positions; therefore, maps are not to physical scale.

Supplementary Tables

A) Summary characteristics of the four GWAS datasets used in this study

**GWAS**	**French**	**German**	**UK**	**USA**
**Cases (GBM/non-GBM)**	1,423 (430/993)	846 (431/415)	631 (270/361)	1,247 (652/595)
Study or Centre	Paris	Bonn	INTERPHONE	MD Anderson
Average age	48	51	46	47
Male/Female	816/607	474/372	394/237	771/476
Illumina Chip	HumanHap 660	HumanHap 660	HumanHap 610 Quad	HumanHap 610 Quad
**Controls**	1,190	1,310	2,699	2,236
Study	SU.VI.MAX	KORA, POPGEN & Heinz Nixdorf Recall	1958 Birth Cohort	CGEMs
Illumina Chip	HumanHap 660	HumanHap 550	Human 1M Duo	HumanHap 240+300; HumanHap 500

B) Details of imputation

GWAS	French	German	UK	USA
**Imputation software**	IMPUTEv2			
**Reference panels**	1000 Genomes Project (1,092 individuals, Phase 1 integrated release version 3), UK10K cohort (3,781 individuals, 2012-06-02 release)			
**Association analysis**	SNPTEST + META			
**Size of inference panel**	425,190			
**SNPS imputed***				
All	12,871,635	13,162,915	11,427,443	13,317,150
MAF >0.5%	8,520,499	8,624,585	8,427,548	8,526,605
MAF <1%	5,102,766	5,282,022	3,708,327	5,533,481
MAF 1-5%	2,070,205	2,103,955	2,022,204	2,062,418
MAF >5%	5,698,557	5,776,844	5,696,827	5,721,165

Supplementary Table 1: Summary characteristics of the four GWAS datasets and details of imputation. Sample numbers are post-QC (as described previously[1,2]). A) Summary characteristics of the four GWAS datasets, B) Details of imputation. * Passing filters of Information score > 0.7 and hardy-weinberg equilibrium $P > 1x10^{-5}$ in controls. MAF, minor allele frequency.

Supplementary Table 2: Best association signals from previously reported glioma GWAS loci discovered in European populations.

							All Glioma		GBM		Non-GBM	
Subtype	Lead SNP	Alleles	Is	Locus	Published SNP	LD (r^2/D')	*P*	OR (95% CI)	*P*	OR (95% CI)	*P*	OR (95% CI)
	rs1920116*	G/**A**	-	3q26.2	rs1920116	-	0.088	0.95 (0.89-1.01)	0.179	0.94 (0.87-1.03)	0.166	0.95 (0.88-1.02)
All glioma	rs10069690	C/**T**	0.78	5p15.33	rs2736100	0.25/0.90	$3.65x10^{-20}$	1.40 (1.30-1.50)	$3.00x10^{-23}$	1.63 (1.48-1.79)	$7.59x10^{-7}$	1.25 (1.15-1.37)
GBM	rs72709458	C/**T**	0.82	5p15.33	rs2736100	0.10/1.00	$4.35x10^{-19}$	1.41 (1.31-1.52)	$6.28x10^{-24}$	1.68 (1.52-1.86)	$7.52x10^{-6}$	1.24 (1.13-1.36)
Non GBM	rs10069690	C/**T**	0.78	5p15.33	rs2736100	0.25/0.90	$3.65x10^{-20}$	1.40 (1.30-1.50)	$3.00x10^{-23}$	1.63 (1.48-1.79)	$7.59x10^{7}$	1.25 (1.15-1.37)
All glioma	rs59060240	T/**TA**	0.86	7p11.2	rs11979158	-	$2.28x10^{-8}$	0.78 (0.72-0.85)	$4.48x10^{-9}$	0.71 (0.63-0.80)	$3.38x10^{-3}$	0.85 (0.77-0.95)
GBM	rs59060240	T/**TA**	0.86	7p11.2	rs11979158	-	$2.28x10^{-8}$	0.78 (0.72-0.85)	$4.48x10^{-9}$	0.71 (0.63-0.80)	$3.38x10^{-3}$	0.85 (0.77-0.95)
Non GBM	rs11974185	A/**T**	0.99	7p11.2	rs11979158	1.00/1.00	$3.16x10^{-8}$	0.80 (0.74-0.87)	$1.77x10^{-8}$	0.74 (0.67-0.82)	$2.09x10^{-3}$	0.86 (0.78-0.95)
All glioma	rs75061358	T/**G**	0.90	7p11.2	rs2252586	0.14/0.80	$2.39x10^{-11}$	1.42 (1.28-1.57)	$5.57x10^{-11}$	1.59 (1.39-1.83)	$2.86x10^{-6}$	1.36 (1.20-1.55)
GBM	rs75061358	T/**G**	0.90	7p11.2	rs2252586	0.14/0.80	$2.39x10^{-11}$	1.42 (1.28-1.57)	$5.57x10^{-11}$	1.59 (1.39-1.83)	$2.86x10^{-6}$	1.36 (1.20-1.55)
Non GBM	rs75061358	T/**G**	0.90	7p11.2	rs2252586	0.14/0.80	$2.39x10^{-11}$	1.42 (1.28-1.57)	$5.57x10^{-11}$	1.59 (1.39-1.83)	$2.86x10^{-6}$	1.36 (1.20-1.55)
All glioma	rs55705857	A/**G**	0.75	8q24	rs4295627	0.16/1.00	$6.01x10^{-37}$	2.19 (1.94-2.47)	$5.65x10^{-3}$	1.28 (1.08-1.53)	$1.18x10^{-62}$	3.60 (3.10-4.19)
GBM	rs57966090	G/**GA**	0.90	8q24	rs4295627	-	$2.76x10^{-8}$	0.83 (0.78-0.89)	$2.12x10^{-4}$	0.85 (0.78-0.93)	$6.93x10^{-7}$	0.81 (0.75-0.89)
Non GBM	rs55705857	A/**G**	0.75	8q24	rs4295627	0.16/1.00	$6.01x10^{-37}$	2.19 (1.94-2.47)	$5.65x10^{-3}$	1.28 (1.08-1.53)	$1.18x10^{-62}$	3.60 (3.10-4.19)
All glioma	rs2157719	T/**C**	-	9p21.3	rs4977756	0.75/0.96	$2.89x10^{-17}$	1.28 (1.21-1.35)	$5.44x10^{-12}$	1.31 (1.21-1.41)	$1.69x10^{-11}$	1.27 (1.19-1.36)
GBM	rs145929329	A/**ATT**	0.80	9p21.3	rs4977756	0.93/1.00	$7.37x10^{-15}$	1.29 (1.21-1.38)	$5.03x10^{-12}$	1.35 (1.24-1.47)	$4.60x10^{-8}$	1.25 (1.15-1.35)
Non GBM	rs2157719	T/**C**	-	9p21.3	rs4977756	0.75/0.96	$2.89x10^{-17}$	1.28 (1.21-1.35)	$5.44x10^{-12}$	1.31 (1.21-1.41)	$1.69x10^{-11}$	1.27 (1.19-1.36)
All glioma	rs12803321	G/**C**	0.98	11q23.3	rs498872	0.18/1.00	$2.20x10^{-16}$	0.78 (0.73-0.82)	0.054	0.93 (0.85-1.00)	$4.33x10^{-25}$	0.68 (0.63-0.73)
GBM	rs1790192	G/**A**	0.79	11q23.3	rs498872	0.09/0.36	$3.23x10^{-6}$	0.86 (0.80-0.91)	0.020	0.90 (0.83-0.98)	$3.58x10^{-6}$	0.83 (0.76-0.90)
Non GBM	rs12803321	G/**C**	0.98	11q23.3	rs498872	0.18/1.00	$2.20x10^{-16}$	0.78 (0.73-0.82)	0.054	0.93 (0.85-1.00)	$4.33x10^{-25}$	0.68 (0.63-0.73)
All glioma	rs35850753	C/**T**	0.87	17p13.1	rs78378222	0.62/1.00	$8.98x10^{-20}$	2.53 (2.07-3.08)	$3.91x10^{-12}$	2.79 (2.09-3.72)	$3.05x10^{-16}$	2.92 (2.26-3.78)
GBM	rs35850753	C/**T**	0.87	17p13.1	rs78378222	0.62/1.00	$8.98x10^{-20}$	2.53 (2.07-3.08)	$3.91x10^{-12}$	2.79 (2.09-3.72)	$3.05x10^{-16}$	2.92 (2.26-3.78)
Non GBM	rs8753	C/**T**	0.89	17p13.1	rs78378222	0.33/0.74	$2.02x10^{-19}$	2.50 (2.05-3.06)	$3.97x10^{-10}$	2.50 (1.88-3.34)	$8.06x10^{-18}$	3.09 (2.39-4.00)
All glioma	rs2236507	C/**G**	0.98	20q13.33	rs6010620	0.95/0.99	$9.26x10^{-11}$	0.79 (0.74-0.85)	$1.57x10^{-13}$	0.70 (0.64-0.77)	$2.63x10^{-3}$	0.88 (0.80-0.96)
GBM	rs6062302	C/**T**	0.98	20q13.33	rs6010620	1.00/1.00	$1.00x10^{-10}$	0.79 (0.74-0.85)	$1.22x10^{-13}$	0.70 (0.64-0.77)	$2.97x10^{-3}$	0.88 (0.81-0.96)
Non GBM	rs139919499	CT/**C**	0.90	20q13.33	rs6010620	-	$2.65x10^{-6}$	1.37 (1.20-1.56)	$1.02x10^{-4}$	1.42 (1.19-1.70)	$5.41x10^{-4}$	1.33 (1.13-1.57)

Supplementary Table 2: Best association signals from previously reported glioma GWAS loci discovered in European populations. For loci with a genome-wide significant *P* value (i.e. < $5x10^{-8}$), the most associated SNPs within the LD block for all glioma as well as GBM and non-GBM subtypes are reported. Linkage disequilibrium (LD) metrics are with respect to the initial reported and published SNPs at 3q26.2, 5p15.33, 7p11.2, 8q24, 9p21.3, 11q23.3, 17p13.1 and 20q13.33[1-4]. * No genome-wide significant variants found in region, therefore reporting SNP as published. CI, confidence interval; OR, odds ratio.

SNP	Is	Status	N	AA concordance		AB concordance		BB concordance		r^2
rs11196067	0.99	Case	187	97.62%	(82/84)	96.59%	(85/88)	100.00%	(18/18)	0.98
		Control	182	96.49%	(55/57)	98.00%	(97/99)	100.00%	(28/28)	0.98
rs12230172	1.00	Case	191	100.00%	(64/64)	100.00%	(93/93)	100.00%	(34/34)	1.00
		Control	182	98.25%	(56/57)	98.70%	(76/77)	96.00%	(48/50)	0.95
rs12780046	0.88	Case	177	95.70%	(133/139)	86.05%	(37/43)	100.00%	(1/1)	0.83
		Control	166	97.12%	(135/139)	87.10%	(27/31)	NA	(0/0)	0.84
rs648044	0.87	Case	150	89.29%	(50/56)	91.36%	(74/81)	95%	(19/20)	0.90
		Control	143	93.44%	(57/61)	85.71%	(60/70)	72.73%	(16/22)	0.86
rs78543262	0.79	Case	180	98.78%	(162/164)	88.89%	(16/18)	NA	(0/0)	0.88
		Control	176	100.00%	(165/165)	100.00%	(11/11)	NA	(0/0)	1.00
rs141035288	0.86	Case	191	89.53%	(171/191)	0%	(0/17)	0%	(0/3)	NA
		Control	183	92.17%	(153/166)	53.33%	(16/30)	0%	(0/1)	0.51
rs76178334	0.89	Case	191	98.88%	(176/178)	86.67%	(13/15)	NA	(0/0)	0.86
		Control	183	100.00%	(174/174)	100.00%	(9/9)	NA	(0/0)	1.00
rs117527984	0.96	Case	191	97.35%	(184/189)	28.57%	(2/7)	NA	(0/0)	0.28
		Control	183	97.56%	(160/164)	81.82%	(18/22)	100.00%	(1/1)	0.82
rs4432939	0.99	Case	192	100.00%	(149/149)	100.00%	(39/39)	100.00%	(4/4)	1.00
		Control	183	99.24%	(130/131)	97.78%	(44/45)	100.00%	(8/8)	0.98
rs182521816	0.74	Case	176	98.28%	(171/174)	40.00%	(2/5)	NA	(0/0)	0.32
		Control	181	99.43%	(173/174)	87.50%	(7/8)	NA	(0/0)	0.87
rs138170678	0.80	Case	188	97.34%	(183/188)	0.00%	(0/5)	NA	(0/0)	0.00
		Control	172	94.15%	(161/171)	9.09%	(1/11)	NA	(0/0)	0.09
rs145034266	0.72	Case	157	97.95%	(143/146)	78.57%	(11/14)	NA	(0/0)	0.77
		Control	173	97.52%	(157/161)	75.00%	(12/16)	NA	(0/0)	0.72

Supplementary Table 3: Imputation quality scores and concordance between directly sequenced and imputed genotype AA, major homozygote; AB, heterozygote; BB, minor homozygote; Is, Information score giving an indication of imputation quality; N, number of comparisons with imputed genotype probability > 90%. r^2 indicates Pearson product momentum correlation coefficient between imputed and sequenced genotype.

Supplementary Table 4: Association between genotype and glioma risk for candidate SNPs selected for further investigation.

					MAF				All Glioma		GBM		Non-GBM	
**SNP**	**Locus**	**Alleles**	**Is**	**Type**	**Case**	**Control**	**Status**	**Study**	***P***	**OR (95% CI)**	***P***	**OR (95% CI)**	***P***	**OR (95% CI)**
rs141035288	3p14.3	A/**G**	0.86	Imputed	0.0015	0.011	1	Discovery	1.70x10^{-13}	0.25 (0.17-0.37)	3.69x10^{-7}	0.32 (0.20-0.49)	6.57x10^{-8}	0.30 (0.20-0.47)
rs117527984	4p15.31	A/**T**	0.96	Imputed	0.0067	0.014	1	Discovery	8.48x10^{-7}	0.50 (0.38-0.66)	2.67x10^{-3}	0.58 (0.41-0.83)	4.30x10^{-5}	0.50 (0.36-0.70)
rs76178334	4q13.3	C/**G**	0.89	Imputed	0.012	0.0086	3	Discovery	7.17x10^{-4}	1.70 (1.25-2.32)	7.74x10^{-7}	2.79 (1.86-4.19)	0.56	1.13 (0.75-1.71)
								Replication	0.55	1.15 (0.73-1.81)	0.51	1.18 (0.71-1.96)	0.47	1.24 (0.69-2.23)
								Combined	1.73x10^{-3}	1.50 (1.16-1.94)	2.05x10^{-5}	1.99 (1.45-2.73)	0.37	1.17 (0.83-1.64)
rs4432939	5q13.1	A/**G**	0.99	Imputed	0.15	0.17	3	Discovery	1.81x10^{-6}	0.83 (0.77-0.90)	1.09x10^{-5}	0.80 (0.72-0.88)	1.93x10^{-3}	0.86 (0.79-0.95)
								Replication	0.84	1.01 (0.89-1.16)	0.76	1.03 (0.88-1.20)	0.97	1.00 (0.83-1.20)
								Combined	5.16x10^{-5}	0.87 (0.82-0.93)	4.50x10^{-4}	0.85 (0.79-0.94)	5.28x10^{-3}	0.89 (0.82-0.97)
rs182521816	6q16.3	A/**G**	0.74	Imputed	0.013	0.0084	3	Discovery	3.95x10^{-5}	2.00 (1.44-2.79)	4.11x10^{-7}	3.07 (1.99-4.75)	0.034	1.65 (1.04-2.63)
								Replication	0.23	0.73 (0.43-1.22)	0.55	0.84 (0.47-1.49)	0.37	0.71 (0.34-1.50)
								Combined	4.71x10^{-3}	1.50 (1.13-1.98)	2.31x10^{-4}	1.92 (1.36-2.72)	0.19	1.30 (0.88-1.94)
rs138170678	9p22.3	A/**T**	0.80	Imputed	0.0022	0.012	1	Discovery	5.89x10^{-13}	0.25 (0.17-0.37)	7.68x10^{-7}	0.32 (0.20-0.50)	6.04x10^{-8}	0.29 (0.19-0.46)
rs145034266	9q22.31	C/**T**	0.72	Imputed	0.021	0.015	2	Discovery	9.91x10^{-4}	1.53 (1.19-1.97)	8.39x10^{-9}	2.70 (1.93-3.79)	0.82	0.96 (0.69-1.33)
rs12780046	10q24.2	G/**T**	0.88	Imputed	0.11	0.09	3	Discovery	7.77x10^{-7}	1.29 (1.17-1.43)	7.65x10^{-3}	1.21 (1.05-1.39)	1.28x10^{-6}	1.37 (1.21-1.55)
								Replication	0.84	1.03 (0.88-1.20)	0.86	0.98 (0.82-1.18)	0.38	1.10 (0.89-1.36)
								Combined	1.49x10^{-5}	1.21 (1.11-1.32)	0.043	1.12 (1.00-1.25)	4.03x10^{-6}	1.29 (1.16-1.44)
rs11196067	10q25.2	A/**T**	0.99	Imputed	0.38	0.41	3	Discovery	6.67x10^{-7}	0.86 (0.81-0.91)	0.06	0.93 (0.86-1.00)	1.54x10^{-8}	0.81 (0.76-0.87)
								Replication	0.56	0.97 (0.88-1.07)	0.91	0.99 (0.89-1.11)	0.33	0.93 (0.81-1.07)
								Combined	4.32x10^{-6}	0.89 (0.85-0.93)	0.11	0.95 (0.89-1.01)	**4.32x10^{-8}**	**0.84 (0.79-0.89)**
rs648044	11q23.2	C/**T**	0.87	Imputed	0.4	0.38	3	Discovery	1.41x10^{-3}	1.11 (1.04-1.18)	0.41	1.04 (0.95-1.13)	3.42x10^{-8}	1.24 (1.15-1.34)
								Replication	0.97	1.08 (0.97-1.19)	0.59	0.97 (0.86-1.09)	4.16x10^{-4}	1.29 (1.12-1.48)
								Combined	5.29x10^{-4}	1.10 (1.04-1.16)	0.32	0.97 (0.90-1.03)	**6.26x10^{-11}**	**1.25 (1.17-1.34)**
rs12230172	12q21.2	G/**A**	1.00	Imputed	0.45	0.46	3	Discovery	4.44x10^{-3}	0.92 (0.87-0.97)	0.76	1.01 (0.94-1.09)	4.82x10^{-6}	0.85 (0.79-0.91)
								Replication	1.84x10^{-6}	0.78 (0.70-0.86)	7.00x10^{-3}	0.85 (0.76-0.96)	3.59x10^{-8}	0.67 (0.58-0.77)
								Combined	1.57x10^{-6}	0.88 (0.84-0.93)	0.22	0.96 (0.91-1.02)	**7.53x10^{-11}**	**0.81 (0.76-0.86)**

					MAF				All Glioma		GBM		Non-GBM	
**SNP**	**Locus**	**Alleles**	**Is**	**Type**	**Case**	**Control**	**Status**	**Study**	***P***	**OR (95% CI)**	***P***	**OR (95% CI)**	***P***	**OR (95% CI)**
rs12230172	12q21.2	G/**A**	1.00	Imputed	0.45	0.46	3	Discovery	$4.44x10^{-3}$	0.92 (0.87-0.97)	0.76	1.01 (0.94-1.09)	$4.82x10^{-6}$	0.85 (0.79-0.91)
								Replication	$1.84x10^{-6}$	0.78 (0.70-0.86)	$7.00x10^{-3}$	0.85 (0.76-0.96)	$3.59x10^{-8}$	0.67 (0.58-0.77)
								Combined	$1.57x10^{-6}$	0.88 (0.84-0.93)	0.22	0.96 (0.91-1.02)	$\mathbf{7.53x10^{-11}}$	**0.81 (0.76-0.86)**
rs3851634	12q23.3	T/**C**	-	Typed	0.27	0.3	3	Discovery	$6.02x10^{-6}$	0.86 (0.81-0.92)	$1.62x10^{-7}$	0.80 (0.74-0.87)	0.039	0.92 (0.85-1.00)
								Replication	0.022	0.88 (0.79-0.98)	$5.00x10^{-3}$	0.83 (0.74-0.95)	0.57	0.96 (0.83-1.11)
								Combined	$4.07x10^{-7}$	0.87 (0.82-0.92)	$\mathbf{3.02x10^{-9}}$	**0.81 (0.76-0.87)**	0.037	0.93 (0.87-1.00)
rs1801591	15q24.2	G/**A**	-	Typed	0.10	0.09	3	Discovery	$5.41x10^{-5}$	1.21 (1.11-1.34)	0.27	1.08 (0.94-1.23)	$1.29x10^{-7}$	1.38 (1.22-1.55)
								Replication	0.16	1.13 (0.95-1.33)	0.89	1.01 (0.83-1.23)	0.013	1.31 (1.06-1.63)
								Combined	$2.75x10^{-5}$	1.20 (1.10-1.30)	0.32	1.06 (0.95-1.18)	$\mathbf{5.71x10^{-9}}$	**1.36 (1.23-1.51)**
rs78543262	19q12	A/**G**	0.79	Imputed	0.018	0.013	3	Discovery	$5.12x10^{-5}$	1.72 (1.32-2.23)	0.37	1.19 (0.82-1.73)	$6.22x10^{-8}$	2.46 (1.77-3.40)
								Replication	0.53	0.90 (0.63-1.26)	0.83	0.96 (0.65-1.41)	0.58	0.87 (0.54-1.42)
								Combined	$4.40x10^{-3}$	1.35 (1.10-1.67)	0.62	1.07 (0.82-1.40)	$2.88x10^{-5}$	1.78 (1.36-2.33)

Supplementary Table 4: Association between genotype and glioma risk for candidate SNPs selected for further investigation. Odds ratios derived with respect to the minor allele, highlighted in bold. MAF, minor allele frequency in discovery series. Shown are discovery association *P* values from meta-analysis of four GWAS datasets, as well as replication and combined meta-analysis *P* values for SNPs taken forward for replication genotyping. Status indicates whether SNP taken forward for replication genotyping (1, not taken forward for replication as poor concordance between imputed and sequenced genotype; 2, not taken forward for replication as repetitive region prevented design of successful genotyping primers; 3, taken forward for replication). Associations in the combined meta-analysis reaching genome-wide significance (i.e. $P < 5x10^{-8}$) are highlighted in bold.

Supplementary Table 5: Genomic annotation of rs648044, rs11196067, rs1801591, rs3851634 and rs12230172.

Locus	SNP	Pos (hg19)	r^2	D'	GERP	CADD	Gene	Annotation	RegulomeDB	FANTOM5	Super-enhancer
10q25.2	**rs11196067**	114469065	1.00	1.00	0.72	4.79	*VTI1A*	intronic	2b	enhancer_tss	
	rs10885375	114475189	0.94	1.00	2.16	8.20	*VTI1A*	intronic	6		
15q24.2	rs17456573	76476361	0.81	0.94	-1.47	3.14	*C15orf27*	intronic	5		
	rs79495512	76492337	0.89	0.95	-3.72	2.45	*C15orf27*	intronic	5		brain_cingulate_gyrus
	rs77815174	76514896	0.91	0.95	0.56	10.71	*C15orf27*	intronic	5		
	rs77387260	76537476	0.92	0.97	3.61	10.59	*ETFA*	intronic	6		
	rs77633900	76538459	0.92	0.97	0.23	3.97	*ETFA*	intronic	NA		
	rs2456074	76555599	0.83	1.00	0.11	1.19	*ETFA*	intronic	4		
	rs2469539	76567484	0.83	1.00	0.5	4.15	*ETFA*	intronic	5		
	rs2469538	76570143	0.83	1.00	0.23	1.56	*ETFA*	intronic	NA		
	rs8042255	76571610	0.83	1.00	0.15	4.07	*ETFA*	intronic	NA		
	rs2469214	76574628	0.84	1.00	1.21	2.85	*ETFA*	intronic	NA		
	**rs1801591**	76578762	1.00	1.00	5.65	29.90	*ETFA*	missense	3a		
	rs2291449	76579234	1.00	1.00	-4.11	4.09	*ETFA*	intronic	5		
	rs2456067	76581418	0.84	1.00	-0.84	0.26	*ETFA*	intronic	5		
	rs2456066	76587371	0.83	1.00	1.43	5.63	*ETFA*	intronic	5		
	rs74805465	76594595	0.98	1.00	-1.3	1.75	*ETFA*	intronic	6		
	rs59673486	76594917	0.83	1.00	NA	NA	*ETFA*	intronic	NA		
	rs2456063	76595549	0.83	1.00	0.17	1.02	*ETFA*	intronic	6		
	rs144886912	76595860	0.83	1.00	NA	NA	*ETFA*	intronic	5		
	rs2956875	76599041	0.83	1.00	1.28	0.35	*ETFA*	intronic	NA		
	rs2469211	76599892	0.83	1.00	-0.98	1.49	*ETFA*	intronic	NA		
	rs2460158	76600222	0.83	1.00	0.95	3.24	*ETFA*	intronic	6		
	rs2469573	76601049	0.83	1.00	-1.42	2.14	*ETFA*	intronic	NA		
	rs2469212	76601075	0.83	1.00	2.78	8.73	*ETFA*	intronic	NA		
	rs78185702	76610235	0.97	1.00	-1.87	0.02	6.4kb 5' of *ETFA*	intergenic	5		
	rs2469215	76611933	0.80	1.00	0.86	0.76	8.1kb 5' of *ETFA*	intergenic	NA		

Locus	SNP	Pos (hg19)	r^2	D'	GERP	CADD	Gene	Annotation	RegulomeDB	FANTOM5	Super-enhancer
	rs6495200	76611934	0.80	-1.00	-1.71	0.20	8.1kb 5' of *ETFA*	intergenic	NA		
	rs2469216	76613477	0.80	1.00	0.09	1.12	9.7kb 5' of *ETFA*	intergenic	5		
	rs2469552	76615481	0.80	1.00	0.5	0.41	12kb 5' of *ETFA*	intergenic	NA		
12q33.33	**rs3851634**	106812902	1.00	1.00	2.37	9.61	*POLR3B*	intronic	5	enhancer_tss	
	rs11112997	106842558	0.89	0.97	1.85	4.23	*POLR3B*	intronic	NA	enhancer_tss	
12q21.2	rs140387126	76233187	0.88	0.99	NA	NA	*RP11-114H23.1*	intronic	6	enhancer_tss	
	rs12426679	76237987	0.88	1.00	-3.27	2.85	*RP11-114H23.1*	intronic	4	enhancer_tss	
	rs12426691	76238046	0.88	1.00	-1.36	4.92	*RP11-114H23.1*	intronic	4	enhancer_tss	
	rs11180710	76238406	0.89	1.00	-6.71	2.69	*RP11-114H23.1*	intronic	6	enhancer_tss	
	rs1565765	76238855	0.89	1.00	-0.98	2.74	*RP11-114H23.1*	intronic	NA	enhancer_tss	
	rs1463754	76239432	1.00	1.00	-0.66	3.80	*RP11-114H23.1*	intronic	3a	enhancer_tss	
	rs1493795	76239977	1.00	1.00	1.54	5.42	*RP11-114H23.1*	intronic	NA	enhancer_tss	
	rs11180712	76240731	1.00	1.00	-5.86	0.86	*RP11-114H23.1*	intronic	NA	enhancer_tss	
	rs11180713	76240797	1.00	1.00	-1.24	3.69	*RP11-114H23.1*	intronic	NA	enhancer_tss	
	rs10879991	76241678	1.00	1.00	-0.03	4.05	*RP11-114H23.1*	intronic	5	enhancer_tss	
	rs10879992	76241681	1.00	1.00	1.25	4.92	*RP11-114H23.1*	intronic	5	enhancer_tss	
	rs10879993	76242047	1.00	1.00	-0.82	11.05	*RP11-114H23.1*	intronic	5	enhancer_tss	
	**rs12230172**	76242675	1.00	1.00	3.41	18.76	*RP11-114H23.1*	intronic	5	enhancer_tss	
	rs12227868	76242802	1.00	1.00	0.53	2.39	*RP11-114H23.1*	intronic	5	enhancer_tss	
	rs1493794	76243379	1.00	1.00	1.57	7.17	*RP11-114H23.1*	intronic	NA	enhancer_tss	
	rs11180714	76243801	1.00	1.00	0.38	4.42	*RP11-114H23.1*	intronic	5	enhancer_tss	
	rs35080799	76244541	1.00	1.00	1.01	6.38	*RP11-114H23.1*	intronic	4	enhancer_tss	
	rs12229980	76245026	1.00	1.00	1.99	7.91	*RP11-114H23.1*	intronic	4	enhancer_tss	u87_GBM_cells
	rs34439598	76245089	1.00	1.00	0.34	1.93	*RP11-114H23.1*	intronic	5	enhancer_tss	u87_GBM_cells
	rs11180719	76245517	0.99	1.00	2.12	7.32	*RP11-114H23.1*	intronic	5	enhancer_tss	u87_GBM_cells
	rs1493792	76246953	0.99	1.00	-2.49	9.70	*RP11-114H23.1*	intronic	5	enhancer_tss	u87_GBM_cells
	rs1493791	76246959	0.99	1.00	2.91	12.30	*RP11-114H23.1*	intronic	5	enhancer_tss	u87_GBM_cells
11q23.2	**rs648044**	114030799	1.00	1.00	-9.32	6.87	*ZBTB16*	intronic	2b	enhancer_promoter	Brain (all)

Supplementary Table 5: Genomic annotation of rs648044, rs11196067, rs1801591, rs3851634 and rs12230172. Data are shown for sentinel SNPs and their proxies (r^2 > 0.8 in 1000 Genomes EUR Phase 1 data) with RegulomeDB scores reflecting evidence of histone marks, DNAse hypersensitivity sites or transcription factor occupancy. Also indicated are genomic evolutionary rate profiling (GERP) scores and combined annotation dependent depletion (CADD) scores as well as overlap with FANTOM5 enhancers[5] and super-enhancers as predicted by Hnisz et al[6]. Chr, chromosome; LD, linkage disequilibrium; Pos, position; tss, transcription start site. RegulomeDB scores: 2b, TF binding + any motif + Dnase Footprint + Dnase peak; 4, TF binding + DNAse peak; 5, TF binding or Dnase peak; 6, other binding or Dnase peak.

SNP	Correlation with rs648044 (r^2/D')	Chr:position	Genotype count (AA/AB/BB)	AA	AB	BB	P_{trend}
**rs648044**	-	11:114,030,799	43/141/98	717	802	936	5.72×10^{-5}
**rs500629**	0.52/0.85	11:114,045,560	45/164/180	698	804	866.5	0.0020
**rs503434**	0.45/0.74	11:114,052,449	71/187/125	707	831	868	3.03×10^{-4}
**rs619151**	0.47/0.84	11:114,061,414	29/145/173	698	755	864	0.0035
**rs526497**	0.50/0.89	11:114,071,287	35/140/143	690	790	865	0.0014
**rs661374**	0.43/0.74	11:114,040,325	68/182/130	700.5	835	866	2.65×10^{-4}

(Columns AA, AB, BB, P_{trend} are under the spanning header *ZW10* expression.)

Supplementary Table 6: Association between rs648044 proxy genotype and *ZW10* expression level in LGG tumours. Genotypes of SNPs correlated with rs648044 (r^2 > 0.4) were assessed for association with *ZW10* expression in low grade glioma (LGG) TCGA tumours by the Kruskal-Wallis trend test. Median expression counts per genotype are given, with the risk allele for each SNP underlined. Chr, chromosome.

Gene	LGG (286 cases)				GBM (273 cases)			
	**Mutation**	**Deletion**	**Amplification**	**Total**	**Mutation**	**Deletion**	**Amplification**	**Total**
VTI1A (10q25.2)	0	6	0	6	0	0	0	0
ZBTB16 (11q23.2)	0	0	3	3	0	0	2	2
PHLDA1 (12q21.2)	0	0	0	0	1 (P370L)	1	2	4
POLR3B (12q23.3)	3 (R768C 2*L372F)	3	1	7	1 (D483N)	0	0	1
ETFA (15q24.2)	0	2	1	3	0	0	0	0
PHLDA1 and *POLR3B*	0	2	1	3	0	0	0	0
POLR3B and *ZBTB16*	0	0	0	0	0	0	1	1
**TOTAL**	**3**	**13**	**6**	**22**	**2**	**1**	**6**	**8**

Supplementary Table 7: Somatic alterations in low grade glioma (LGG) and GBM tumours from the cancer genome atlas. The cBioPortal was interrogated for carriers of mutations, deletions and amplifications affecting target genes in a sample set of 286 low grade glioma (LGG) and 273 GBM TCGA samples.

				All glioma		GBM		Non GBM	
**Locus**	**SNP**	**Control MAF**	**Reference**	**Relative risk per minor allele**	**% of total variance in risk explained**	**Relative risk per minor allele**	**% of total variance in risk explained**	**Relative risk per minor allele**	**% of total variance in risk explained**
**Previously reported loci**									
5p15.33	rs72709458	0.21	This study	1.41	2.92	1.68	6.65	1.24	1.14
7p11.2	rs59060240	0.16	This study	0.78	1.24	0.71	2.35	0.85	0.53
7p11.2	rs75061358	0.08	This study	1.42	1.36	1.59	2.37	1.36	1.04
8q24	rs55705857	0.06	[7]	2.50	7.31	1.30	0.60	4.30	18.53
9p21.3	rs2157719	0.43	This study	1.28	2.25	1.31	2.69	1.28	2.25
11q23.3	rs12803321	0.35	This study	0.78	2.11	0.93	0.18	0.68	5.09
17p13.1	rs78378222	0.02	[8]	3.74	4.40	5.59	7.49	5.01	6.57
20q13.3	rs6062302	0.23	This study	0.79	1.46	0.70	3.34	0.88	0.43
		**TOTAL**			**23.04**		**25.67**		**35.58**
**Loci first reported in this study**									
10q25.2	rs111696067	0.41	This study	0.89	0.49	0.95	0.10	0.84	1.11
11q23.2	rs648044	0.38	This study	1.10	0.32	0.97	0.03	1.25	1.76
12q21.2	rs12230172	0.46	This study	0.88	0.61	0.96	0.06	0.81	1.66
15q24.2	rs1801591	0.09	This study	1.20	1.05	1.06	0.11	1.36	2.98
12q23.3	rs3851634	0.30	This study	0.79	1.46	0.70	3.34	0.88	0.43
		**TOTAL**			**2.71**		**0.84**		**7.58**
		**OVERALL**			**25.76**		**26.52**		**43.15**

Supplementary Table 8: Individual variance in risk associated with glioma SNPs. For each glioma risk locus, the relative risk per minor allele of the highest associated SNP is given. If the highest-associated SNP in a given locus has been published, relative risks are derived from published studies, otherwise they are derived from this study. MAF, minor allele frequency.

Pathway/gene set name	*P*-value	FDR
**All glioma**		
CELL CYCLE CHECKPOINT GO 0000075	< 0.001	0.0020
G1 TO S CELL CYCLE REACTOME	< 0.001	0.021
HSA00230 PURINE METABOLISM	< 0.001	0.026
CELL CORTEX PART GO:0044448	< 0.001	0.029
HSA00600 SPHINGOLIPID METABOLISM	< 0.001	0.055
ENERGY DERIVATION BY OXIDATION OF ORGANIC COMPOUNDS	0.0010	0.056
REGULATION OF MITOSIS	0.0010	0.057
G1PATHWAY	0.0020	0.060
REGULATION OF CELL CYCLE	< 0.001	0.061
HSA05219 BLADDER CANCER	< 0.001	0.070
ORGANELLE OUTER MEMBRANE	0.0020	0.071
HSA00240 PYRIMIDINE METABOLISM	0.0010	0.075
TRANSCRIPTION INITIATION FROM RNA POLYMERASE II PROMOTER	0.0030	0.077
HSA04110 CELL CYCLE	< 0.001	0.098
**GBM glioma**		
HSA00240 PYRIMIDINE METABOLISM	< 0.001	0.010
HSA00230 PURINE METABOLISM	< 0.001	0.011
HSA04610 COMPLEMENT AND COAGULATION CASCADES	< 0.001	0.072
CELL CELL ADHESION	< 0.001	0.080
HSA04330 NOTCH SIGNALING PATHWAY	< 0.001	0.082
REGULATION OF PHOSPHORYLATION	< 0.001	0.094
MACROMOLECULE CATABOLIC PROCESS	0.0010	0.095
BIOPOLYMER CATABOLIC PROCESS	0.0010	0.095
**Non GBM glioma**		
CELL CYCLE CHECKPOINT GO 0000075	< 0.001	0.015
TRANSCRIPTION INITIATION FROM RNA POLYMERASE II PROMOTER	< 0.001	0.021
G1PATHWAY	< 0.001	0.021
G1 TO S CELL CYCLE REACTOME	0.0020	0.076
REGULATION OF MITOSIS	0.0010	0.083
ENERGY DERIVATION BY OXIDATION OF ORGANIC COMPOUNDS	0.0010	0.083
RNA TRANSCRIPTION REACTOME	0.0010	0.088
MITOCHONDRIAL PART	0.0010	0.095
KERATINOCYTEPATHWAY	0.0010	0.096

Supplementary Table 9: Biological pathways enriched for glioma GWAS association signals. Shown are all pathways with false discovery rate (FDR) < 0.10.

Type	Primer	Sequence (5’-3’)
Genotyping	rs648044_A	GAAGGTGACCAAGTTCATGCTGCTGTTTTCTTCTGCTCAGCA
	rs648044_G	GAAGGTCGGAGTCAACGGATTCTGCTGTTTTCTTCTGCTCAGCG
	rs648044_Common	CTAGCCAGGAAGTGAGAGCCGTT
Sequencing	rs648044_Forward	AGGCTGTGGAGAAGTGAGTC
	rs648044_Reverse	ACAAACACAGGCAATGCTCG
Genotyping	rs1801591_A	GAAGGTGACCAAGTTCATGCTTGTTGCTGCAGCATCAAAGGATA
	rs1801591_G	GAAGGTCGGAGTCAACGGATTGTTGCTGCAGCATCAAAGGATG
	rs1801591_Common	GTGAAGTGTGATGAGAAAGTGAAAGTGTT
Genotyping	rs3851634_C	GAAGGTGACCAAGTTCATGCTGATGAAGAAGCCTATTCCAGCAG
	rs3851634_T	GAAGGTCGGAGTCAACGGATTGGATGAAGAAGCCTATTCCAGCAA
	rs3851634_Common	GATGCTTATTCAAGTTCCTTTTGCCAGTT
Genotyping	rs4432939_T	GAAGGTGACCAAGTTCATGCTGATGCTAACATCTCATTTTCCTTTCCAT
	rs4432939_C	GAAGGTCGGAGTCAACGGATTATGCTAACATCTCATTTTCCTTTCCAC
	rs4432939_Common	CCAAGAGTGACCCTTGAAATGAATTCTA
Sequencing	rs4432939_Forward	GGGCAAAGTGGGAGGATAGT
	rs4432939_Reverse	GCCAGTGTCAACGAGCTA
Genotyping	rs11196067_T	GAAGGTGACCAAGTTCATGCTAGGGTCAAAAATCTTCATTGTATACTCT
	rs11196067_A	GAAGGTCGGAGTCAACGGATTAGGGTCAAAAATCTTCATTGTATACTCA
	rs11196067_Common	AAGTTGGAGCCCTGTATTTTCACAGATAA
Sequencing	rs11196067_Forward	GTCTGGTTGCTTTGCTGACA
	rs11196067_Reverse	GAAGGTAGAGGCTGCATTGC
Genotyping	rs12230172_A	GAAGGTGACCAAGTTCATGCTAAGCCGGCCCCAGAGGCAA
	rs12230172_G	GAAGGTCGGAGTCAACGGATTGCCGGCCCCAGAGGCAG
	rs12230172_Common	GAGCAAGAGCTAGGATTAAGAATTGGAAA
Sequencing	rs12230172_Forward	TTTGATGACTTGCATTTTCAAT
	rs12230172_Reverse	CTGCAAGTTGCTCAGCTCAC
Genotyping	rs12780046_G	GAAGGTGACCAAGTTCATGCTTGTGGAAATTGAGCTAAATTGTTGTG
	rs12780046_T	GAAGGTCGGAGTCAACGGATTAACTTGTGGAAATTGAGCTAAATTGTTGTT
	rs12780046_Common	GGGTATGCGGAAACCTTTGGGTTTA
Sequencing	rs12780046_Forward	TCTCAGTGTACCTCTTCTGGA
	rs12780046_Reverse	AGGGGTATGCGGAAACCTTT
Genotyping	rs76178334_C	GAAGGTGACCAAGTTCATGCTAGAGCTATTCGGGCTCTTGC
	rs76178334_G	GAAGGTCGGAGTCAACGGATTCTAGAGCTATTCGGGCTCTTGG

	rs76178334_Common	CATGGAGATGACATTGAAAGACTGACTAT
Sequencing	rs76178334_Forward<br>rs76178334_Reverse	TGGATGACAGCCAGGGAATC<br>TGTATTGGGCTTTGCAAGGG
Genotyping	rs78543262_A<br>rs78543262_G<br>rs78543262_Common	GAAGGTGACCAAGTTCATGCTACTGTGCAACATAACAAGGTCCCA<br>GAAGGTCGGAGTCAACGGATTCTGTGCAACATAACAAGGTCCCG<br>CATGCATCACCATGCCTGGCTAATT
Sequencing	rs78543262_Forward<br>rs78543262_Reverse	CCACTTAAATTGGTAAACAGGCC<br>CTGCAATCCTCCCACCTCA
Genotyping	rs182521816_A<br>rs182521816_G<br>rs182521816_Common	GAAGGTGACCAAGTTCATGCTAGGCATAACCAGATAAAAAAATAGAGTTCA<br>GAAGGTCGGAGTCAACGGATTGGCATAACCAGATAAAAAAATAGAGTTCG<br>AGACTAATGCTTCTATCATGTCTCCATATT
Sequencing	rs182521816_Forward<br>rs182521816_Reverse	TGGGTATGTGTGCTTGTGTAC<br>GCTCTAAGTCAAATGAATGTGGG
Sequencing	rs117527984_Forward<br>rs117527984_Reverse	TCCCTCTCTAATGAAGCCTGT<br>AGTTAGGACCATGGGCTTGA
Sequencing	rs138170678_Forward<br>rs138170678_Reverse	TGAACTGTATGCTCCTCAAGG<br>GGAGCTTCTAGACATGTTTCAGG
Sequencing	rs141035288_Forward<br>rs141035288_Reverse	GGTCTGCCCGATTTCCAAATT<br>ACGCCATTCTTCTGCCTC
Sequencing	rs145034266_Forward<br>rs145034266_Reverse	CCTGGCTGATTTTGCATTTT<br>GGATTGCATTCCTGGTTTCT

Supplementary Table 10: Genotyping and sequencing primer sequences.

Supplementary References

1. **Sanson, M. *et al.* Chromosome 7p11.2 (EGFR) variation influences glioma risk. *Hum Mol Genet* 20, 2897-904 (2011).**
2. **Shete, S. *et al.* Genome-wide association study identifies five susceptibility loci for glioma. *Nat Genet* 41, 899-904 (2009).**
3. **Wrensch, M. *et al.* Variants in the CDKN2B and RTEL1 regions are associated with high-grade glioma susceptibility. *Nat Genet* 41, 905-8 (2009).**
4. **Walsh, K.M. *et al.* Variants near TERT and TERC influencing telomere length are associated with high-grade glioma risk. *Nat Genet* 46, 731-5 (2014).**
5. **Andersson, R. *et al.* An atlas of active enhancers across human cell types and tissues. *Nature* 507, 455-61 (2014).**
6. **Hnisz, D. *et al.* Super-enhancers in the control of cell identity and disease. *Cell* 155, 934-47 (2013).**
7. **Enciso-Mora, V. *et al.* Deciphering the 8q24.21 association for glioma. *Hum Mol Genet* 22, 2293-302 (2013).**
8. **Enciso-Mora, V. *et al.* Low penetrance susceptibility to glioma is caused by the TP53 variant rs78378222. *Br J Cancer* 108, 2178-85 (2013).**
